# Recent Progress on the Synthesis, Morphological Topography, and Battery Applications of Polypyrrole-Based Nanocomposites

**DOI:** 10.3390/polym16233277

**Published:** 2024-11-25

**Authors:** Mohammad Mizanur Rahman Khan, Md. Mahamudul Hasan Rumon

**Affiliations:** 1 Department of Mechanical Engineering, Gachon University, 1342 Seongnam-daero, Sujeong-gu, Seongnam-si 13120, Gyeonggi-do, Republic of Korea; 2 Department of Chemistry, Indiana University of Bloomington, Bloomington, IN 47405, USA; mdhasa@iu.edu

**Keywords:** conductive polymers, polypyrrole, nanocomposites, synthesis, morphology, electrode material, polymerization approach, and battery applications

## Abstract

Polypyrrole (PPy)-based nanocomposite materials are of great interest to the scientific community owing to their usefulness in designing state-of-the-art industrial applications, such as fuel cells, catalysts and sensors, energy devices, and especially batteries. However, the commercialization of these materials has not yet reached a satisfactory level of implementation. More research is required for the design and synthesis of PPy-based composite materials for numerous types of battery applications. Due to the rising demand for environmentally friendly, cost-effective, and sustainable energy, battery applications are a significant solution to the energy crisis, utilizing suitable materials like PPy-based composites. Among the conducting polymers, PPy is considered an important class of materials owing to their ease of synthesis, low cost, environmentally friendly nature, and so on. In this context, PPy-based nanocomposites may be very promising due to their nanostructural properties and distinct morphological topography, which are vital concerns for their applications for battery applications. Such features of PPy-based nanocomposites make them particularly promising for next-generation electrode materials. However, the design and fabrication of appropriate PPy-based nanocomposites for battery applications is still a challenging area of research. This review paper describes the current progress on the synthesizing of PPy-based composites for battery applications along with their morphological topography. We discussed here the recent progress on the synthesis of different PPy-based composites, including PPy/S, PPy/MnOx, MWCNT/PPy, V_2_O_5_/PPy, Cl-doped PPy/rGO, and Fe/α-MnO_2_@PPy composites, by a polymerization approach for numerous battery applications. The insights presented in this review aim to provide a comprehensive reference for the future development of PPy-based composites in battery technology.

## 1. Introduction

Conducting polymers are regarded by recent research as one of the important areas of study in the field of synthetic polymers because of their superior electrical, mechanical, thermal, morphological, and optical properties and so on [1,2,3,4]. Among the conducting polymers, polypyrrole (PPy) is the most significant one due to its high electrical conductivity [5,6], reversible doping/dedoping feature, simple synthesis [7], and excellent environmental monitoring [8]. Because of these characteristics, it has a wide range of uses, including batteries, sensors, capacitors, [9], electrodes, [10], and more. As a result, composites made of PPy are of great interest because of their inherent electrical, morphological, and low-cost production qualities [11]. The functionality and performance of PPy can be increased by the introduction of other materials. In this way, the scope of applications of PPy-based composites was enhanced by the researchers, as they possess synergy between the individual components from which they are formed [5,12,13]. PPy-based composites, despite their promising properties, face several drawbacks that limit their performance in energy storage applications. Their inherent brittleness and poor mechanical stability hinder their long-term cycling performance, while limited electrical conductivity under certain conditions affects their charge transfer efficiency. Additionally, the structural degradation during repeated charge–discharge cycles impacts their electrochemical stability. To address these challenges, improvements in mechanical robustness, conductivity enhancement, and structural stability are essential, as these directly influence its capacity retention, rate capability, and overall application efficiency. These aspects require further clarification to tailor PPy for specific energy storage systems effectively. Thus, the properties (for example morphology) and application (for example battery) of PPy can be improved by incorporating various filler materials, including carbon nanotubes (CNTs), metals, metal oxides, and so on [11,14].

The introduction of filler materials into PPy to form composites may lead to electronic interactions, morphological features, and a combination of the effects between the constituents of composite materials. For instance, the incorporation of CNTs, ZnO, TiO_2_, and NiO into PPy demonstrated better electrical conductivity, morphology, and chemical stability [11,15,16,17,18]. PPy and metal oxide (MnO_2_, ZnO, TiO_2_, NiO, and V_2_O_5_) composites have been described to have catalytic behavior and high conductivity for use in wastewater treatment and battery applications [17,18,19,20]. The synthesis of PPy-based composites with an effective morphological form for applications in different batteries is still challenging.

Battery applications have become a cornerstone of energy research, addressing the challenges posed by the intermittent and fluctuating nature of renewable energy sources [21,22,23,24]. While renewable energy is being increasingly recognized as a sustainable alternative to fossil fuels, its large-scale adoption is hindered by stability and storage issues. Advanced battery technologies, such as those utilizing innovative materials like PPy, offer promising solutions for efficient energy storage, ensuring a reliable and cost-effective energy supply to meet the growing global demand [25,26,27,28]. Since rechargeable batteries with comparatively less battery efficiency represent a major disadvantage, high safety, affordability, and environmental friendliness are required in this regard [29]. The limitations of the use of traditional battery materials are that they possess relatively poor performance and less durability. To overcome such problems, the design and fabrication of new materials like PPy-based composites may be promising. Specifically, the modification of the cathode and anode utilizing these campsites can be beneficial for battery applications. Further, in regard to the interesting properties of PPy, PPy-based composites are applied for different types of batteries, including sodium-ion, aqueous zinc-ion (AZIBs), potassium ion, lithium-ion, and lithium-sulfur [29,30,31,32,33].

The present review focuses on the recent developments in PPy-based composite materials fabrications for battery applications. The different PPy-based composites for different types of battery applications are also discussed. In addition, we have explained here the recent research work on the numerous polymerization approaches for the synthesis of PPy-based composites and the significant conditions of the fabrication, as well as the morphological topography of these types of materials.

## 2. Synthesis of Polypyrrole (PPy)-Based Nanocomposites

**Figure 1:**
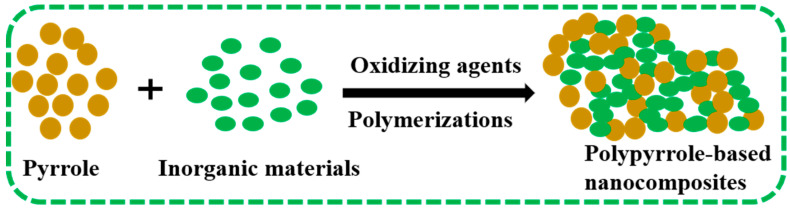
Synthesis of polypyrrole-based nanocomposites [35].

**Figure 2:**
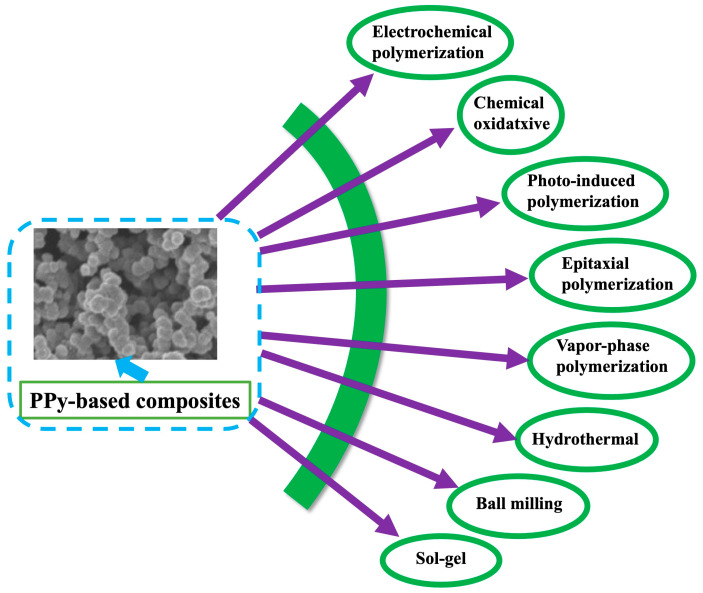
Schematic illustration of the numerous synthesis processes of PPy-based composites.

For the synthesis of PPy-based nanocomposites, PPy and metal (for example, Ag, Au), metal oxides (for example, CuO, CaO), and other inorganic materials (for example, ZnS) have been extensively utilized by the researchers [34,35,36,37,38]. In the synthesis process, pyrrole is allowed to react with the inorganic materials in the presence of oxidizing agents, and following the reaction conditions, polypyrrole-based nanocomposites are formed (Figure 1). For example, An et al. synthesized PPy/carbon nanotube nanocomposites through a chemical oxidative polymerization technique to enhance the sensitivity of a gas sensor [39]. PPy/MnO_2_ nanocomposites were prepared by the electrochemical method in reference [35]. The authors described that the huge surface area of PPy chains facilitates the metal oxides to incorporate into a polymer matrix [40]. The numerous synthesis techniques for the formation of PPy-based composites are displayed in Figure 2 and reviewed in the following subsections:

### 2.1. Electrochemical Polymerization Approach

The electrochemical polymerization technique is widely used for the synthesis of PPy-based composites owing to its numerous advantages such as ease of synthesis, requiring less monomer, low cost, environmental friendliness, and so on [18,41,42,43]. This method is also utilized for the generation of electroactive materials in film form where the properties of the produced films can be controlled by changing the electrolysis parameters including current density, electrode potential, electrolyte, and used solvent for the synthesis process [43,44]. For instance, PPy/MXene composites were fabricated by Jian et al. [45] to utilize as electrode materials, especially for energy applications. In such a process, PPy and Mxene were electrochemically dropped into the substrate either in a way process [46] or in a single step [47]. These works have been performed for the applications in battery based on PPy-based composites, which are shown in Table 1.

### 2.2. Chemical Oxidative Polymerization Techniques

Chemical oxidative polymerization is a simple and convenient approach for the synthesis of PPy-based composite materials [62,63]. This technique is extensively used by researchers owing to its numerous suitable properties such as thermal stability, conductivity, processability, and so on, which are significant for battery applications [62,64]. For instance, Veronika et al. [48] prepared a PPy-sulfur composite through oxidative polymerization for Li-S battery applications. The authors used FeCl_3_ as an oxidizing agent, and the reaction mixture was continuously stirred for 2 h during the synthesis process. Lim et al. [50] prepared polypyrrole-MWCNT core-shell nanowire composites for high-performance rechargeable sulfate- and sodium-ion batteries. The authors used a mixture of 5 M nitric acid and 2 M hydrochloric acid, which were chemically treated for 5 h at 80 °C in the synthesis process.

Huang and his co-workers synthesized PPy-S-coated multi-walled carbon nanotube (MWCNTs@S-PPy) composites using FeCl_3_ as an oxidant, where the prepared composites showed great performances for lithium-sulfur batteries [58]. In the synthesis process, firstly, sulfur was grown on MWCNTs, and the PPy was developed on the external face of MWCNTs@S. In this case, PPy acts as a conductive mattress. Zhang et al. prepared V_2_O_5_-PPy composites by the in situ polymerization technique at room temperature for aqueous zinc-ion batteries [20]. In the synthesis process, the reaction mixture was stirred for 144 h. Huang et al. synthesized PPy/MnO_2_/Mn_2_O_3_ nanocomposites by in situ polymerization for high-performance aqueous zinc-ion batteries [54]. The authors fabricated such composites through ultrasonication and stirring at different times of 0.5 h, 2 h, and 3.5 h at room temperature [54].

Zhu et al. synthesized LiNi_1/3_Co_1/3_Mn_1/3_O_2_/polypyrrole composites for high-efficiency lithium-ion batteries [55]. PPy was fabricated by the chemical oxidative polymerization approach and following an ultrasound dispersion procedure. The prepared LiNi_1/3_Co_1/3_Mn_1/3_O_2_/polypyrrole composites were applied as cathode material [19]. Xue et al. fabricated PPy-modified Prussian blue (KHCF@PPy) composites by the in situ polymerization approach [56]. The prepared KHCF@PPy composites were applied as a cathode material for potassium ion batteries. The polymerization process involved the introduction of K_4_Fe(CN)_6_·H_2_O into the PPy in an acid medium, followed by continuous magnetic stirring for 4 h. Liang et al. prepared PPy@S@PPy composites through chemical oxidative polymerization for lithium-sulfur batteries. The synthesis process took place with continuous stirring for 30 min at 0–5 °C [60].

### 2.3. Photo-Induced Polymerization Techniques

Photo-induced polymerization is suitable for synthesizing PPy and PPy-based composites [62,65,66,67]. This technique is appealing because of its easy processability, environmental friendliness, and better thermal stability [68], which are significant for applying composite materials for batteries. For instance, Xu et al. [51] prepared polypyrrole Fe-coated porous silicon materials for high-performance lithium-ion battery applications. This synthesis technique involved the utilization of acid for etching from SiAl alloy. The authors stated that the fabrication technique is simple and eco-friendly.

### 2.4. Epitaxial Polymerization

In epitaxial polymerization, crystal growth or material deposition occurs when new crystalline layers are formed with one or more well-defined orientations regarding the crystalline seed layer. In this approach, it is possible to enhance the structural strength of the materials through a polymerized structure [49]. Through epitaxial polymerization, Zhang et al. [49] prepared PPy/MnOx nanosheets for zinc ion battery application. This polymerization took place in the liquid phase, which may provide a unique perspective to the preparation of high-performance electrode materials and 2D PPy-based polymer composites [49].

### 2.5. Vapor-Phase Polymerization Approach

The vapor-phase synthesis approach is a suitable technique in which the monomer is provided to a substrate that is oxidant-coated and in vapor form, and polymerization then undergoes at the oxidant and in the vapor interface. Materials accompany the improved electrode surface utilizing the method of vapor polymerization [69]. Thin films are commonly deposited on conductive surface materials using polymerization techniques. Vapor polymerization processes have been employed to apply thin films on flexible substrates such as cotton, wood, and other conductive surfaces. These methods have also been utilized in developing electronic noses and DNA sensors [70,71]. Mohammadi et al. performed the first studies of vapor-phase polymerization of PPy using FeCl_3_ and H_2_O_2_ as oxidizing agents at low pressure [72]. On the other hand, researchers also used normal pressure for vapor-phase polymerization [73,74]. Numerous types of oxidants such as Fe (Tosylate)_3_, CuCl_2_, and HAuCl_3_ are used in vapor-phase polymerization [75,76].

### 2.6. Hydrothermal Method

**Figure 3:**
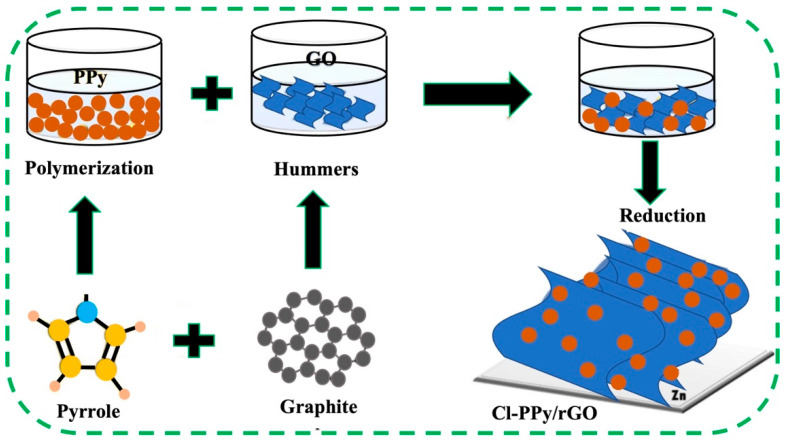
Synthesis of Cl-doped PPy/rGO composites through hydrothermal technique [69].

Liao et al. synthesized 3-D β-MnO_2_/PPy composites for applications in highly efficient cathode in zinc-ion batteries [52]. Qin et al. synthesized V_2_O_5_/PPy composites by the surface-initiated strategy and following the hydrothermal methods for aqueous zinc-ion batteries [52]. In the prepared composites, V_2_O_5_ nanowires were wrapped by PPy. Zhang et al. synthesized oxygen-deficient hydrate vanadium dioxide (HVO) with polypyrrole (O_d_−HVO@PPy) by a one-step hydrothermal process where oxygen vacancy is introduced in HVO during the in situ polymerization [53]. Ju Duan et al. synthesized Cl-doped PPy/rGO composites by the oxidative chemical polymerization process for sodium-based dual-ion batteries [31]. The authors claimed that the straight use of Cl-doped PPy/rGO composites in working electrodes can improve battery energy density and practical capacity [31,73]. The synthesis approach of Cl-doped-PPy/rGO film is represented in Figure 3.

In the polymerization process, firstly, Cl-doped PPy nanoparticles were synthesized by oxidative chemical polymerization using FeCl_3_ as an oxidant, and rGO was produced using Hummers’ process [69,77]. Cl-PPy nanoparticles and GO solution were gradually and proportionately mixed. Cl-doped-PPy/rGO exhibits flexibility and strong structural stability, allowing it to be randomly wrapped and shaped into any desired shape. Feng et al. prepared hierarchical MnO/PPy composites by the hydrothermal process for lithium-sulfur batteries [57]. The preparation process involves ultrasonication and stirring for 30 min in the air, followed by hydrothermal treatment [57].

### 2.7. Ball Milling Process

**Figure 4:**
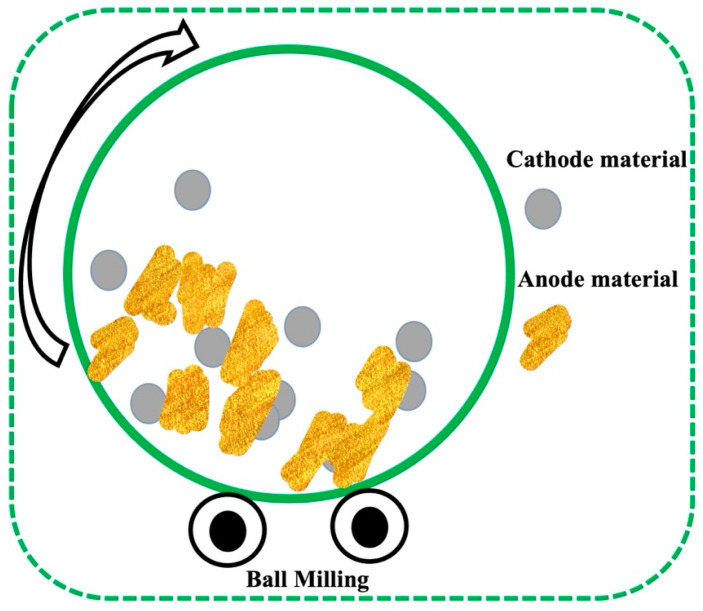
Schematic illustration of the fabrication of S/PPy composite through using one-step ball milling process [69].

The ball milling process is also a convenient technique for the synthesis of PPy-based composite materials. Zhang et al. fabricated nanostructured S/PPy composites utilizing a one-step ball milling process without heat treatment, as presented in Figure 4 [69]. To synthesize S/PPy composites, PPy was blended with S, maintaining the proportion of S: PPy 2:1 by the ball milling process and stirring for 3 h [69,78]. The prepared S/PPy composites have enhanced electrochemical performance in lithium batteries through the enhanced charge transfer in the electrode.

### 2.8. Sol-Gel Method

**Figure 5:**
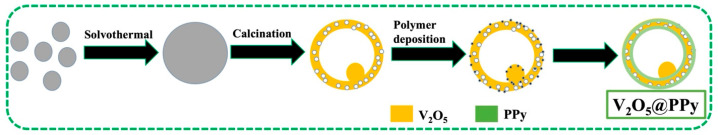
Schematic demonstration of the synthesis of PPy-coated V_2_O_5_ yolk-shell nanospheres [61].

The sol-gel method is a versatile and effective approach for synthesizing PPy-based composite materials. A key advantage of this technique is its ability to produce materials with high surface areas and stable surfaces. For instance, Liang and colleagues synthesized PPy-coated V_2_O_5_ yolk-shell nanospheres using the sol-gel method for V_2_O_5_ preparation. The PPy coating was subsequently achieved through a combination of solvothermal processing and vapor deposition, as illustrated in Figure 5 [61]. The PPy@V_2_O_5_ composites exhibited a great cycling performance of lithium-ion batteries [61,69].

The design and synthesis of PPy-based nanocomposites utilizing numerous synthesis approaches have shown great potential for sensors, energy storage applications, and so on. Nonetheless, their materialistic usage remains a challenge for researchers because PPy itself has numerous drawbacks, including the formation of secondary growth and maintaining its nanostructure by controlling the synthesis at different reaction conditions (for example, sonication, pH, temperature, reaction time, etc.). Further, the addition of dopant materials for the generation of nanocomposites plays a vital role in their electronic and morphological properties. Since PPy has a comparatively shorter shelf-life in doped form, the formation of PPy-based composites with extended life is significant for its large-scale industrial usage. Although considerable work has been provided by scientists to alleviate the limitations for usage, further research is needed regarding directing the design and synthesis to enhance the performances of PPy-based composites for battery applications.

## 3. Morphological Topography

**Figure 6:**
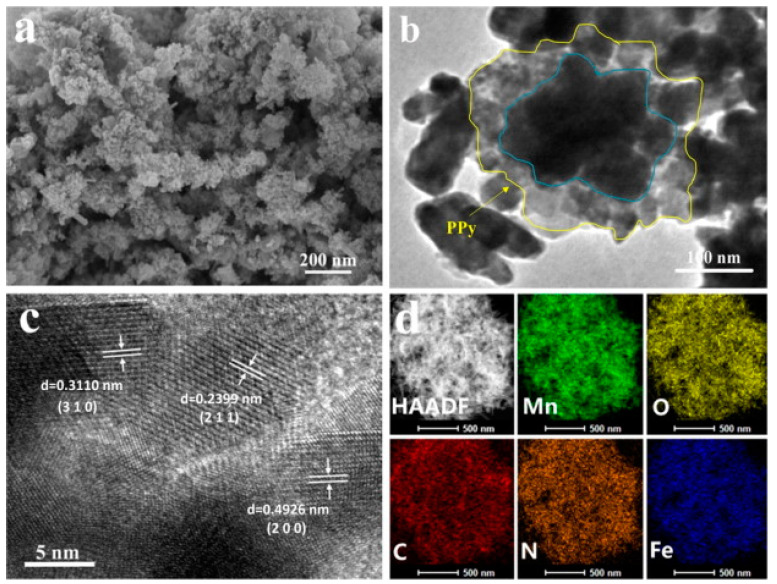
(**a**) SEM micrographs of Fe/a-MnO_2_@PPy composites. (**b**) TEM and (**c**) HRTEM of Fe/a MnO_2_@PPy composites. (**d**) The Mapping images of Mn, O, C, N, and Fe elements [30]. Reproduced with the permission from Ref. [30]. Copyright 2021, Elsevier.

The morphological behavior of the PPy-based composites has a significant impact on the application of these materials in battery areas. Depending on the synthesis approach, the morphology of the PPy-based composites varied, as discussed by the researchers [50,61,63]. For example, the morphological properties of the Fe/a-MnO_2_@PPy composites synthesized by Xu et al., 2021 [30,45] by in situ polymerization are presented in Figure 6.

Figure 6a demonstrates the SEM image where the deposition of PPy on the surface of Fe-doped MnO_2_ is appreciated. The TEM image of Fe/α-MnO_2_@PPy (Figure 6b) reveals a strong interaction between α-MnO_2_ and PPy in the central region, as indicated by the blue and yellow curves. The MnO_2_ particles serve as a template in an acidic medium, facilitating the in-situ polymerization of PPy particles [30]. As shown in Figure 6c, the HRTEM image exhibits lattice fringe spacings of 0.3110, 0.2399, and 0.4926 nm, which correspond to the (3 1 0), (2 1 1), and (2 0 0) planes of iron-doped α-MnO_2_, respectively [30,45]. Further, the EDX elemental compositions of Mn, O, C, N, and Fe corroborate the composite structure (Figure 6d), and the presence of N and C directs the existence of PPy. Similarly, Mn, O, C, N, and Fe are the main elements that match to α-MnO_2_ and concur in the composite [30].

Huang et al. synthesized PPy-S-coated MWCNT composites as cathode materials by the chemical oxidation approach and discussed the morphological characteristics of the prepared materials [58]. The authors demonstrated that MWCNTs demonstrated linear structure with a smooth surface, having a diameter of 30 nm [58]. Liang and his coworkers synthesized split-half-tubular PPy@S@PPy composites of 3D structures as cathode materials for applications in lithium-sulfur batteries [60].

**Figure 7:**
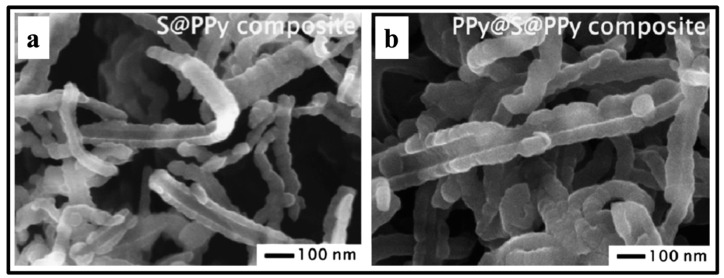
FESEM micrographs of (**a**) S@PPy composite, (**b**) PPy@S@PPy composite [58]. Reproduced with the permission from Ref. [60]. Copyright 2015, Elsevier.

The morphological features of the freshly prepared S@PPy composite and PPy@S@PPy composite are presented in Figure 7. The micrographs of the as-prepared S@PPy composite and PPy@S@PPy composite demonstrated that both of the S@PPy composites and PPy@S@PPy composites have the split-half-tube structure, and the tube-like diameter remains about 100–150 nm tube width, as given in Figure 7a,b [60].

**Figure 8:**
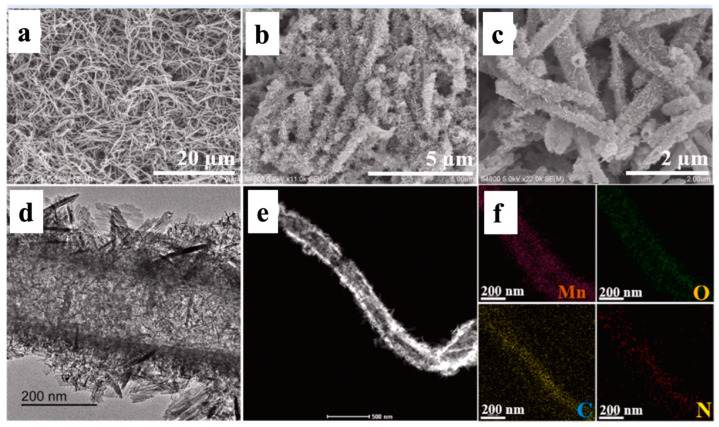
SEM micrograph of (**a**) PPy nanotubes and (**b**,**c**) MnO/PPy composite. (**d**) TEM image, (**e**) STEM micrograph, and corresponding (**f**) EDS mapping micrograph of the MnO/PPy composite [60]. Reproduced with the permission from Ref. [57]. Copyright 2022, Elsevier.

Feng and his co-workers synthesized MnO/PPy composites by hydrothermal process and explained the morphological topography using SEM, TEM, STEM, and EDX measurements, as presented in Figure 7 [57]. Figure 8a represents the SEM micrograph of PPy nanotubes with a diameter of about 250 nm. The MnO/PPy composite demonstrated the SEM micrograph of PPy nanotubes with MnO nanosheets after hydrothermal synthesis, as presented in Figure 8b,c, which is again confirmed by the TEM image shown in Figure 8d.

A STEM micrograph is shown in Figure 8e, and the corresponding EDX data are presented in Figure 8f, where the mapping images imply that the Mn, O, C, and N are uniformly distributed within the MnO/PPy composite [60].

## 4. Battery Applications

PPy-based materials hold significant promise for battery applications, particularly in zinc-ion, sodium-ion, lithium-ion, and lithium-sulfur batteries due to their unique electrochemical properties [79,80,81]. As a conductive polymer, PPy offers excellent electrical conductivity, high mechanical flexibility, and good environmental stability, making it an ideal component for electrode materials [82,83]. In Zn-ion and Na-ion batteries, PPy can enhance ion diffusion and improve cycling stability, addressing key challenges like low energy density and short lifespan [84,85]. In lithium-ion and lithium-sulfur batteries, PPy’s ability to form a conductive matrix helps in mitigating the dissolution of polysulfides in LiS systems and improves Li-ion transport efficiency [86,87]. Additionally, PPy can be easily synthesized and modified, allowing for tailored electrochemical performance, making it a versatile and promising material in the development of next-generation energy storage systems [88]. The PPy-based composite materials for numerous types of battery applications are reviewed in the following subsection.

### 4.1. PPy-Based Composite in Battery Applications

Batteries exhibit strong oxidation potential due to the use of metal oxide cathodes, which can trigger the disintegration process in liquid organic electrolytes [89]. Recent advancements in lithium battery technology have introduced polymer-based separators that replace conventional liquid electrolytes with thin polymer membranes [90]. These membranes serve as pathways for ion transport during battery charge and discharge cycles, primarily acting as a barrier between the cathode and anode while enabling ion conduction.

In electric vehicles, batteries utilizing polymer electrolytes often demonstrate ion conductivities above 10 S/cm within the 20 to 60 °C temperature range [91]. For such applications, polymers must exhibit excellent thermal, mechanical, and electrochemical stability. Solid-state electrochemistry is the foundation for the use of polymer materials as separators in batteries, with conductive polymer films being a key feature in economically viable battery systems [92]. These conductive coatings are vital for reducing direct interactions between cathode and electrolyte materials. For a substrate to be effective in battery applications, it must facilitate reversible electrochemical redox reactions [93]. PPy stands out as an ideal candidate due to its favorable redox properties, environmental stability, cost-effectiveness, high conductivity, and ease of synthesis [94,95]. Chemical processes allow PPy to achieve the desired surface morphology. Furthermore, positive doping techniques can significantly enhance the conductivity of PPy to several tenths of S/cm [63]. As a result, extensive research is ongoing to explore PPy’s potential in diverse battery applications, leading to innovative methods for its integration into battery systems.

#### 4.1.1. Sodium Dual-Ion Batteries (SDIBs)

Sodium-ion batteries (SIBs) have recently gained significant attention from both academic and industrial sectors as a promising alternative to lithium-ion batteries (LIBs) for various applications [96,97]. This interest stems primarily from the abundant availability of sodium resources and the compatibility of SIBs with existing electrolyte systems used in LIBs. However, the larger ionic radius of sodium ions (1.020 Å) compared to lithium ions (0.720 Å) presents challenges for electrode materials during the repeated processes of sodium ion insertion and extraction [98]. This issue is particularly critical for cathode materials, which play a vital role in defining the power density and lifespan of SIBs. Consequently, sluggish sodium kinetics and subpar cycling performance hinder the overall efficiency of SIBs [99].

While traditional SIBs operate by cyclically introducing and extracting Na^+^ between the anode and cathode during charging and discharging, sodium dual-ion batteries (SDIBs) adopt a different approach [100,101]. SDIBs incorporate anions (such as ClO4 and PF6) from the electrolyte alongside Na^+^ into both the anode and cathode, marking a significant shift from conventional SIB mechanisms [102]. SDIBs are emerging as strong candidates in the electronics industry. The cathode materials employed in SDIBs offer economic and environmental advantages, utilizing organic compounds, sodium metal, and alloy-type anodes that deliver higher specific capacities compared to traditional cathode materials [103,104]. Notably, in 2021, Ju Duan and his team investigated PPy/reduced graphene oxide composites, employing a straightforward hydrothermal method to synthesize free-standing and flexible chlorine-doped PPy/rGO [29]. This innovative composition enhances charge transfer and accelerates oxidation-reduction reactions, leading to improved overall capacity. Additionally, the effective dispersion of electrons and ions results in significantly elevated battery energy density and practical capacity. This research not only stipulates a simple technique for developing high-performance cathodes for SDIBs but also clarifies the role of redox-active polymers in facilitating ion storage.

The synthesis begins with the production of chlorine-doped PPy nanoparticles via oxidative chemical polymerization, while reduced graphene oxide (rGO) is generated using Hummers’ method [105,106]. The doping process employs ferric chloride as both a dopant and an oxidizing agent. The GO solution is then blended with Cl-PPy nanoparticles in a controlled manner [69]. Following this, zinc foil is immersed in the mixture, leading to the formation of free-standing chlorine-doped PPy/rGO through self-assembly and simultaneous reduction. The flexibility and structural integrity of Cl-doped PPy/rGO allow it to be shaped into diverse forms.

The resulting Cl-PPy/rGO demonstrates impressive electrical discharge capacities of 0.422 Ah/g at 100 mA/g, an extraordinary rate capacity of 0.148 Ah/g at 500 mA/g, and a noteworthy reversible capacity of 0.183 Ah/g at 0.1 Ah/g when coupled with a hard carbon anode. Moreover, the composite’s ion storage mechanism was examined through a combination of cyclic voltammetry, ex situ X-ray photoelectron spectroscopy, and Fourier-transform infrared spectroscopy. This study not only presents a straightforward method for creating superior cathodes for SDIBs but also elucidates the ion storage mechanism in redox-active conductive polymers [85,107]. Additionally, Zhu Limin and colleagues explored lithium-based cathode materials coated with PPy, demonstrating improved lithium-ion diffusion and reduced charge transfer resistance [55].

#### 4.1.2. Potassium Ion Batteries (PIBs)

Nowadays, potassium-ion batteries (PIBs) have emerged as an attractive alternative to LIBs for large-scale energy storage, thanks to the abundant availability of potassium resources and the similar electrochemical characteristics shared by lithium and potassium. However, the larger ionic radius of K^+^ (1.38 Å) compared to Li^+^ (0.76 Å) can lead to unfavorable phase transformations and volume expansions in traditional inorganic cathodes during K^+^ insertion and extraction, adversely affecting cycle stability and limiting PIB performance [108]. To address this challenge, researchers are exploring various alternatives, with conducting polymers (CPs) showing significant promise due to their structural versatility and excellent mechanical stability.

In 2021, Ju Duan and his research group developed a PPy and carbon nanotube (CNT) nanocomposite (1D-PPy-coated-CNT) using a single-step oxidative polymerization method to serve as a cathode material for PIBs [109]. This composite features a one-dimensional structure with a uniform layer of PPy coating on the carbon nanotube surface. The CNT facilitates electron and ion transport, while the PPy layer enhances ion storage and increases active sites. Over 200 cycles, the PPy-coated-CNT exhibits an exceptional discharge capacity of 0.148 Ah/g at 100 mA/g, retaining 76% of its capacity, marking a significant advancement in potassium-ion batteries. The integration of the PPy coating improves the ion transport rates and introduces additional reaction sites, leading to enhanced capacity [31]. The PPy-coated CNT showcases an excellent reversible capacity of 0.113 Ah/g after 200 cycles at 100 mA/g. The ion storage mechanism of 1D PPy-coated CNT offers a promising new pathway for cathode materials in PIBs, generating substantial interest in enhancing performance and utilizing PPy for energy storage [106,110,111].

The findings from these studies underscore the potential of PPy as a high-performance cathode material for potassium-based dual-ion batteries. Prussian blue and its analogs are recognized as competitive cathodes for PIBs due to their open structures and modifiable frameworks [112]. However, the inherent lattice defects and low electrical conductivity of Prussian blues can lead to subpar cycling performance and rate capabilities. Researchers are implementing various strategies to address these challenges, including morphological modification, surface enhancement, nanostructure design, and compositional optimization [113,114]. Qing Xue and his team introduced an in-situ polymerization coating technique for PPy-modified Prussian blue material [59]. This conductive polymer significantly enhances the rate capability of KHCF by boosting its electronic conductivity, resulting in outstanding rate capability and cycling stability. The impressive cycling stability may be attributed to the retention of K ions within the monoclinic structure, as evidenced by comprehensive characterization, which also disclosed the nonexistence of phase transitions during the charging and discharging processes.

#### 4.1.3. Aqueous Zinc-Ion Batteries (AZIBs)

Aqueous zinc-ion batteries (AZIBs) are gaining attention as a promising option for large-scale energy storage due to their affordability and environmental benefits [115]. However, the development of AZIBs is currently hindered by a lack of efficient cathode materials. Many existing cathodes face challenges such as low ion diffusion rates, cathode material dissolution, and poor electronic conductivity [115]. Manganese-based cathode materials are particularly attractive due to their sustainability and cost-effectiveness, yet they suffer from poor electrical conductivity, a tendency for manganese ion dissolution, and structural instability, all of which hinder their electrochemical performance [116]. To overcome these limitations, there is a need to design high-performance cathode materials using MnO_2_ and its composite forms. MnO_2_ composites incorporating PPy have shown significant potential due to their unique structural properties and polymorphic phases, including the λ, β, δ, α, and γ types [117]. Among these, α-MnO_2_ is notable for its extensive 2 × 2 tunnel structures, which enhance zinc-ion reaction kinetics during cycling. However, α-MnO_2_ is susceptible to structural degradation over repeated charge–discharge cycles [118,119]. Conversely, β-MnO_2_ demonstrates improved thermodynamic stability due to its compact (1 × 1) channel structure and exposed (101) layers, providing greater resilience [19]. Nanorods of α-MnO_2_ exhibit a discharge capacity of 0.12 Ah/g at a current density of 0.52 A/g, reaching a maximum specific capacity of 0.270 Ah/g at 0.10 A/g, while also demonstrating remarkable cycling stability. Nevertheless, β-MnO_2_ electrodes often encounter higher resistance to charge transfer and slower reaction kinetics, limiting their cathode performance. Various strategies have been explored to enhance the effectiveness of manganese oxide electrodes, such as improving the electrolyte, incorporating conductive materials, and utilizing cation doping. Among these strategies, the integration of conductive materials has proven particularly effective in boosting cathode performance.

**Figure 9:**
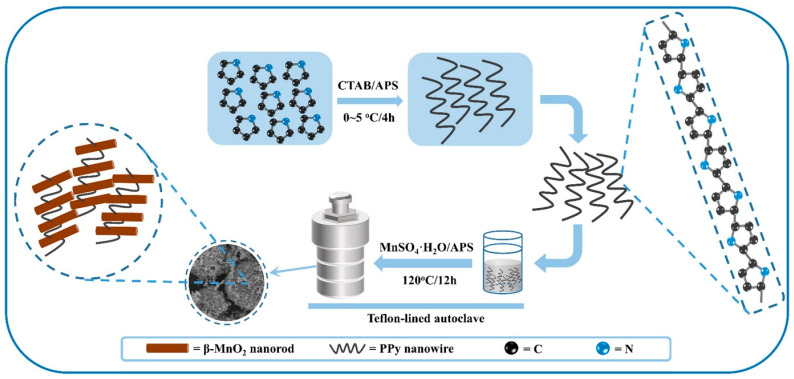
Schematic representation of the two-step synthesis process for the PPy-modified-β-MnO_2_ nanocomposite. This figure is adopted from Ref. [19]. Copyright 2017, Elsevier.

Recent progress has focused on the development of PPy-coated β-MnO_2_ hybrid materials to enhance cathode effectiveness in Zn-ion batteries. The synthesis of these composite materials involves a two-step process [19]. First, PPy nanowires are prepared via oxidative polymerization, followed by the hydrothermal fabrication of the PPy-coated β-MnO_2_ composites. When employed as cathodes, these β-MnO_2_/PPy composites demonstrate a significant specific discharge capacity of 0.36 Ah/g at a current density of 0.200 A/g, outperforming unmodified MnO_2_ powder. The enhanced electrochemical performance of PPy-modified β-MnO_2_ is attributed to its 3D mesoporous microsphere architecture and the superior conductivity of PPy, which is formed through the assembly of PPy nanowires and α-MnO_2_ nanorods, as shown in Figure 9.

Xiaoling Zang and his research team explored manganese-based cathode materials for aqueous zinc-ion batteries (AZIBs) due to their eco-friendliness and cost-effectiveness [49]. They successfully developed a high-performance cathode by synthesizing two-dimensional manganese oxide-coated PPy (2D MnOx/PPy) through a specific polymerization method. This technique involved the epitaxial polymerization of PPy around two-dimensional manganese oxide nanosheets, utilizing sodium dodecyl sulfate (SDS) as an inhibitor. The manganese oxide nanosheets are inherently amorphous and are often encapsulated with PPy due to interactions between Mn and N atoms. The experimental results revealed exceptional performance, attributed to the two-dimensional morphology that enhances structural integrity and facilitates both electron and ion conductivity [49]. The MnOx/PPy composite achieved an impressive discharge capacity of 0.40 Ah/g at a 1 Coulomb rate and maintained a capacity retention of 78% after 2800 cycles at a 5 Coulomb rate, demonstrating considerably enhanced rate capability and capacity compared to pure MnO_x_. This outstanding performance is likely due to the advantages of the simultaneous epitaxial polymerization of the 2D polymeric material. Importantly, this study confirms that the two-dimensional morphology of PPy-coated MnOx remains largely intact during long-term testing. Electrochemical simulations further indicate enhanced ion diffusion rates and reduced electron transfer resistance in the manganese oxide-coated PPy (MnOx/PPy) compared to both pure PPy and manganese oxide.

In 2021, considerable attention was directed toward PPy cathodes for AZIBs. For instance, Zhang Zhengchunyu et al. synthesized oxygen-deficient hydrate vanadium dioxide with a PPy coating (Od-HVO@PPy) using a single hydrothermal phase, concurrently creating oxygen vacancies in HVO during in situ polymerization [53]. Additionally, Huang Aixiang et al. developed a manganese dioxide/manganese trioxide nanocomposite to enhance electronic conductivity and reduce the degradation of manganese-based cathodes [54]. They utilized self-initiated polymerization and molten salt synthesis to apply a thin layer of PPy onto the MnO_2_/Mn_2_O_3_ nanocomposite. The PPy-coated MnO_2_/Mn_2_O_3_ nanocomposite achieved a specific capacity of 0.289 Ah/g at a 200 mA/g rate and maintained a capacity of 0.199 Ah/g at 3000 mA/g, demonstrating a remarkable cycling stability of 97% after 1000 cycles at 1 A/g. The results revealed minimal electrochemical polarization, reduced post-cycling charge transfer impedance, a significant contribution from capacitance, and a high electrolyte ion mass transfer coefficient. The liquid-phase epitaxial polymerization of conducting polymers presents a novel approach to creating materials suitable for high-performance electrodes and two-dimensional conducting polymers.

**Figure 10:**
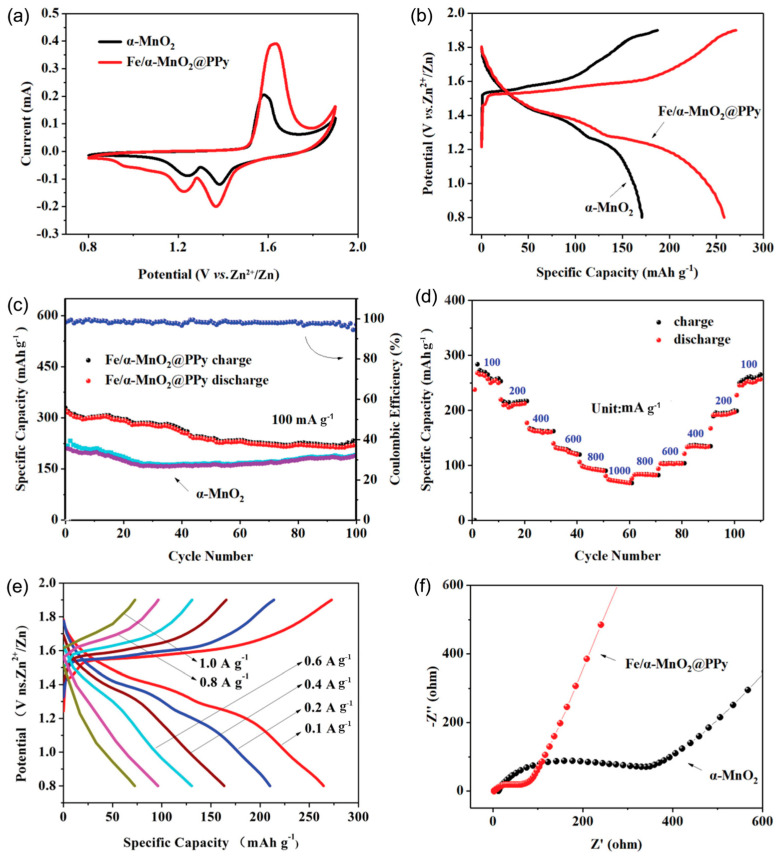
Schematic illustration for the comparison of the electrochemical performance of Fe/α-MnO_2_@PPy and α-MnO_2_ electrodes. (**a**) Cyclic voltammetry curves. (**b**) Charge/discharge performance. (**c**) Cycling stability and (**d**) rate capability. (**e**) Charge/discharge profiles at varying current densities. (**f**) Electrochemical impedance spectroscopy (EIS) plots for the cathodes. Reproduced with the permission from Ref. [30]. Copyright 2021, Elsevier.

Jun-Wei Xu et al. investigated a cathode composed of iron-doped alpha manganese dioxide for AZIBs [30]. To fabricate the PPy-coated Fe-doped α-MnO_2_ composite cathode, the team initially synthesized the Fe-doped α-MnO_2_ using a chemical precipitation method, followed by polymerization of the pyrrole monomer initiated with HCl to form the PPy coating, as shown in Figure 10. The PPy composite was collected via centrifugation. The incorporation of iron enhanced the lattice spacing of α-MnO_2_, promoting the rapid accumulation of zinc ions. The PPy coating facilitates structural adjustments in MnO_2_, reinforcing both its stability and electron conductivity. The synergistic effects of PPy doping and coating are believed to improve the performance of MnO_2_. The PPy-coated Fe-doped α-MnO_2_ composite exhibited a specific capacity of 0.27 Ah/g after 100 cycles at 0.1 A/g and a coulomb efficiency close to 99%. This innovative design of the PPy-coated Fe-doped α-MnO_2_ composite holds significant promise for zinc-ion batteries, indicating enhanced performance due to the PPy coating and iron doping, which aid in the design and development of PPy-coated Fe-doped α-MnO_2_ composites for AZIB cathodes [30]. To address challenges such as low ion diffusion coefficients, cathode dissolution, and inadequate electronic conductivity, Yan Zhang et al. focused on the V_2_O_5_-PPy composite for AZIBs [20]. They employed in situ polymerization at ambient temperature to create V_2_O_5_-PPy nanobelt composites on a large scale, with V_2_O_5_ serving as an oxidant for pyrrole polymerization. Their findings demonstrated that the synthesized V_2_O_5_-PPy nanobelt composite significantly improved electronic conductivity, mitigated vanadium dissolution, and facilitated the formation of oxygen vacancies.

Additionally, Xinghua Qin and his team explored PPy-wrapped V_2_O_5_ nanowires for AZIBs [52]. They synthesized the PPy-coated V_2_O_5_ nanowire through a surface-initiated polymerization strategy, leveraging the redox reaction between V_2_O_5_ and pyrrole. The PPy coating not only enhanced electronic conductivity but also minimized the dissolution of V_2_O_5_. The V_2_O_5_/PPy composite cathode demonstrated a high specific capacity of 466 mAh/g at 0.1 A/g and exceptional cycling stability, retaining 95% of its capacity after 1000 cycles at a high current density of 5 A/g. Dong Ruichen et al. investigated a cathode material composed of V_2_O_5_ particles encased in a PPy layer (V_2_O_5_/PPy), produced through environmentally friendly methods [120]. Their research indicated that the PPy coating significantly enhances cycling and rate performance. The resulting V_2_O_5_/PPy cathode exhibited a high-rate capacity of 68.4 mAh/g at 5 A/g, along with a remarkable capacity retention of 95.6% after 300 cycles.

#### 4.1.4. Lithium-Ion Batteries (LIBs)

Lithium-ion batteries (LIBs) are recognized as efficient energy storage solutions for portable devices, such as smartphones, laptops, and electric vehicles due to their high energy density (ED), mechanical strength, and long cycle life [121,122]. However, safety concerns arise when LIBs operate outside standard conditions, such as during mechanical impacts, short circuits, or overheating, which can lead to thermal hazards and gas generation [123]. To mitigate these risks, various strategies have been developed, including advanced battery management methods like accurate state-of-charge and temperature estimation using ultrasonic reflection waves, as well as rapid identification of micro-health parameters. These methods complement chemical modifications to enhance the performance and safety of LIBs. Additionally, PPy-based composites offer a promising solution for improving battery safety and efficiency, bridging the gap between material innovation and effective battery management. For instance, Yun-Hui Huang et al. improved the electrochemical performance of LIBs by coating lithium iron phosphate (LiFePO_4_) cathodes with PPy [124]. The carbon-coated LiFePO_4_/PPy thin film was synthesized using in situ electrodeposition, and electrochemical evaluations were performed using CR2032 coin cells. LiFePO_4_ is a preferred cathode material due to its theoretical capacity (0.170 Ah/g), environmental sustainability, and stable voltage profile, but it suffers from low electronic conductivity and limited Li^+^ diffusion, hindering its high-rate capabilities. PPy, with its high conductivity and porous structure, addresses these limitations by enhancing the cathode’s electronic and ionic conductivities. Similarly, Yuan Gao et al. synthesized LiFePO_4_/PPy nanorod composites and demonstrated that a 2.95% PPy coating significantly improved the material’s discharge capacity (0.153 Ah/g) and reduced charge transfer resistance [125]. This improvement was attributed to the uniform PPy coating that facilitated efficient Li^+^ diffusion, especially at low temperatures, making the material promising for use in electric vehicles (EVs) and hybrid electric vehicles (HEVs).

**Figure 11:**
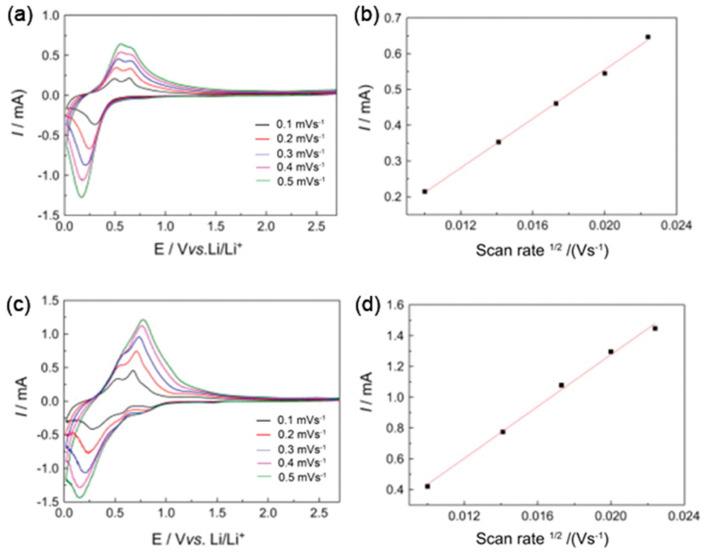
Cyclic voltammetry profiles for hollow SnO_2_ microspheres (**a**) and hollow SnO_2_@PPy (21 wt%) core-shell nanocomposite anode (**c**) at scan rates of 0.1, 0.2, 0.3, 0.4, and 0.5 mV s^−1^. Corresponding plots of peak current (Ip) as a function of the square root of scan rate (v^1/2^) for hollow SnO_2_ microspheres (**b**) and hollow SnO_2_@PPy (21 wt%) nanocomposite anode (**d**) are presented. Reproduced with permission from Ref. [128]. Copyright 2017, Elsevier.

LIBs are increasingly preferred over traditional battery technologies like nickel-cadmium and lead-acid batteries due to their lightweight, high-energy density, and extended cycle life [126]. However, materials like tin (Sn)-based anodes, which offer high theoretical capacity, face challenges such as significant volume changes during lithium alloying/dealloying and poor conductivity. Nanoscale designs and hybrid materials have been developed to overcome these issues. For instance, Cui et al. reported that SnO_2_/PPy composites demonstrated superior Li-ion diffusion and charge/discharge performance due to the mesoporous PPy film, which alleviated the stress from volume changes [127]. Liu et al. fabricated hollow SnO_2_/PPy nanocomposites, which exhibited excellent cycling performance and a larger diffusion length [128]. The hollow SnO_2_ structure allowed for volumetric changes, while the PPy coating prevented nanoparticle aggregation and fragmentation (Figure 11). In another study, SnO_2_/rGO/PPy ternary anodes achieved over 117.6 mAh/g at 3900 mA/g with enhanced rate performance and stability [129]. Reduced graphene oxide (rGO) and PPy synergistically improved electron transfer and reduced strain during charge/discharge cycles. Similarly, Zhao et al. developed a coaxial nanocable composed of single-walled carbon nanotubes (SWNTs), SnO_2_, and PPy, which delivered excellent electrochemical performance (823 mAh/g after 100 cycles) by combining high conductivity and volume control [130]. Wang et al. explored a triaxial nanocable made of PPy, SnS_2_, and carbon nanofibers (CNFs) that demonstrated remarkable lithium storage capacity due to its hierarchical structure, which facilitated electrolyte diffusion and reduced charge transfer resistance [131]. Given the geographical limitations and cost of lithium, alternative materials like sodium and sulfur are being explored for battery applications. Spinel ferrites (AFe_2_O_4_, where A = Zn, Ni, Co, Mn), integrated with PPy, have emerged as promising alternatives. For example, CoFe_2_O_4_/PPy nanotubes developed by He et al. demonstrated excellent electrochemical performance and sodium-ion diffusion, suggesting potential for sodium-ion batteries [132]. However, V_2_O_5_ is widely recognized as an effective cathode material in lithium batteries due to its high valence state and layered structure. However, its electrochemical performance is often hampered by the chemical breakdown of vanadium peroxide in electrolytes, limiting its cyclic longevity. Xing Liang et al. investigated innovative PPy-coated vanadium pentoxide yolk-shell nanospheres synthesized via a sol-gel technique [61]. Initially, V_2_O_5_ was created and then enveloped in PPy through a combination of solvothermal methods and vapor deposition. The PPy layers, formed via chemical oxidative polymerization, exhibited remarkable hydrophobic properties, efficiently retaining moisture within the electronic materials. For electrochemical evaluations, the electrode was prepared by blending V_2_O_5_, PVDF, and carbon black in N-methyl pyrrolidone, adhering to a weight ratio of 0.7:0.2:0.1. An analysis revealed that V_2_O_5_/PPy demonstrates exceptional cycling stability, effectively reducing the chemical degradation of V_2_O_5_ while significantly enhancing electrical conductivity. At discharge current densities of 0.10 A/g and 0.5 A/g, the material achieved a prolonged cycling life of 1000 cycles and 5000 cycles (with a specific discharge capacity of 0.121 Ah/g) within a voltage range of 2.50–4.10 V. The PPy-coated V_2_O_5_ nanosphere thus exhibits outstanding cycling capabilities for lithium-ion batteries (LIBs). This proposed method offers a novel strategy to mitigate the degradation of active materials within the electrolyte, providing an innovative approach to protect these critical compounds from dissolution.

In contrast, traditional lithium-ion batteries utilizing LiCoO_2_ cathodes present safety hazards and frequently struggle to meet the escalating energy storage demands for electric vehicles (EVs) [133]. Elevating operational voltage is a key strategy for improving energy and power densities in Li-ion batteries. Currently, LiNiMnCoO_2_ (LNMO) is hailed as a promising cathode material for LIBs due to its high voltage characteristics [134]. Nevertheless, elevated temperatures can lead to the dissolution of manganese and nickel, resulting in adverse reactions that hinder the practical application of LNMO in lithium-ion batteries. To address this challenge, researchers have proposed implementing a metal oxide layer to create a protective barrier of conductive material, thereby reducing the dissolution of manganese and nickel. PPy is frequently chosen for its cost-effectiveness and ease of achieving desired morphologies. Xuanwen Gao and his team explored PPy-coated NMO composites to enhance the electrochemical characteristics of LIBs [60]. Their study involved fabricating micron-sized LNMO with a PPy coating through chemical oxidative polymerization in an aqueous medium. The process began with the production of MnO_3_ microspheres via a standard reaction, followed by filtration to collect the white precipitate. The bare LNMO was then synthesized, and the LNMO-PPy composite was formed through straightforward chemical oxidative polymerization. The findings indicated that various inorganic materials, such as Bi_2_O_3_, ZnO, Al_2_O_3_, and Co_3_O_4_, function primarily as protective layers for the surface of the reactive material without enhancing the rated capacity of LNMO. In contrast, the integration of PPy proved effective in improving electron transfer in LNMO, significantly boosting its electrochemical performance. Notably, LNMO with 5 wt.% PPy exhibited superior Coulombic efficiency and cycling stability compared to bare LNMO. Chemical analyses of the lithium foil anode revealed that the PPy coating effectively mitigated the dissolution of nickel and manganese in LNMO. Additionally, the PPy layer protects the cathode from degradation products originating from the electrolyte at elevated temperatures, resulting in improved Coulombic efficiencies. Their results suggest that the LNMO-PPy composite serves as an advanced cathode material for LIBs with enhanced energy and power characteristics, leveraging PPy’s protective capabilities to prevent electrolyte degradation caused by detrimental interactions at the surface of the active material.

In another insightful study conducted by Mi-Ra Shin et al., Mi-Ra Shin and Jong-Tae Son investigated a modified external layer of LiNi_0.6_Co_0.2_Mn_0.2_O_2_ (LNMO) cathodes using PPy, achieved through a blending process in a dispersion medium [135]. Ethanol and xylene served as dispersing solvents, yielding a porous coating layer with ethanol and a dense coating with xylene. This surface modification resulted in a 15 nm thick layer of PPy on LNMO. The initial discharge capacity of the PPy-coated LNMO synthesized with ethanol exhibited excellent electrochemical characteristics, achieving a discharge capacity of 162.4–161.5 mAh/g. The charge transfer resistances for the PPy-coated LNMO and bare electrodes synthesized with ethanol were measured at 119.3 and 432.1 Ω, respectively. The lithium diffusion coefficients of the bare and ethanol-synthesized PPy-coated LNMO electrodes were determined to be 4.20 × 10^−16^ and 2.25 × 10^−14^ cm^2^/s, respectively. This study concluded that the ethanol-synthesized PPy-coated LNMO cathode offers a greater lithium diffusion coefficient, improved electrochemical performance, and reduced charge transfer barriers compared to bare electrodes, attributed to the porous PPy surface facilitating lithium movement away from the underlying electrode. Differential scanning calorimetry (DSC) measurements indicated that the ethanol-synthesized PPy-coated LNMO electrode generated less heat than its bare counterpart.

Lihuan Xu et al. examined composite electrodes derived from a nitroxide free radical-containing pyrrole copolymer, utilizing a self-synthesized TEMPO (2,2,6,6-tetramethylpiperidinyl-N-oxy) containing pyrrole as a functional monomer to create a TEMPO-containing pyrrole copolymer [136]. The active material was initially synthesized through electrochemical polymerization and subsequently used as a cathode for lithium-ion batteries. The morphology, structure, charge–discharge performance, and electrochemical characteristics of the synthesized PPy were characterized using scanning electron microscopy (SEM), Fourier-transform infrared spectroscopy (FTIR), and electrochemical methods. The TEMPO-containing PPy copolymer exhibited an enhanced specific capacity of 0.07 Ah/g. Two samples were prepared: one composite based on PPy-co-PPy-C-TEMPO with a 4:1 ratio and another with an 8:1 ratio. The 4:1 ratio demonstrated a regular spherical structure combined with smaller particles of approximately 400 nm, promoting electron network connectivity. The 8:1 ratio exhibited a discharge capacity of 0.062 Ah/g, while the 4:1 ratio reached approximately 0.070 Ah/g. Consequently, the PPy-co-PPy-C-TEMPO-based (4:1) composite displayed superior cell performance and discharge capacity compared to neat PPy (PPy), attributed to the incorporation of TEMPO into the PPy backbone and the use of in situ electrochemical polymerization methods to establish the initial morphology. This research positions the TEMPO-containing PPy copolymer as a promising high-performance cathode material for lithium-ion batteries.

Xiaoran Sun and his team explored PPy-coated zinc ferrite hollow spheres (ZnFe_2_O_4_/PPy) using a spray-drying technique to produce double-shelled hollow spheres of ZnFe_2_O_4_ [137]. The vapor-phase polymerization method was employed to coat the zinc ferrite hollow spheres with PPy. Initially, micron-sized precursor spheres were created through spray drying, followed by exposing zinc ferrite hollow nanospheres to vapor-phase PPy coating. Utilizing ferric chloride (FeCl_3_) as a catalyst, the pyrrole monomers were in situ polymerized into PPy, resulting in PPy-coated ZnFe_2_O_4_. During this polymerization, the color of the zinc ferrite hollow nanospheres transitioned from orange to black, indicating successful coating with PPy. This PPy layer enhances the structural stability and electrical conductivity of ZnFe_2_O_4_. When used as an anode in LIBs, the PPy-coated zinc ferrite hollow spheres demonstrated improved cycling stability and rate capability compared to bare ZnFe_2_O_4_ hollow spheres. This innovative system has the potential to serve as an anode material with extensive capacity for materials like silicon, which often experience low conductivity and significant volumetric changes. The internal volume accommodates alterations during charge/discharge cycles, while the PPy layer enhances structural integrity. The results indicate that ZnFe_2_O_4_-PPy displays enhanced cycling stability, which can fortify structural integrity and elevate the performance of the PPy-coated zinc ferrite composite across various charge/discharge currents from 0.1 to 3.20 mA/g. Lingna Sun et al. focused on NiCo_2_O_4_/PPy composites to enhance performance. They synthesized NiCo_2_O_4_ particles via a hydrothermal method and applied a PPy coating through chemical oxidative polymerization of pyrrole [138]. By varying the concentrations of the pyrrole monomer, they produced different compositions of NiCo_2_O_4_.

#### 4.1.5. Lithium-Sulfur Batteries (LiSBs)

Rechargeable lithium-sulfur batteries (LiSBs) have garnered significant attention in recent years due to several compelling advantages [139]. Sulfur, known for its abundant availability and cost-effectiveness, offers an impressive theoretical capacity, positioning LiSBs as promising candidates for applications in electric vehicles and large-scale energy storage solutions [140]. With a remarkable energy density of up to 2600 Wh kg^−1^, Li-S batteries provide a much more appealing alternative to conventional lithium-ion batteries, which typically achieve only 387 Wh kg^−1^ [141]. Despite their advantages, Li-S batteries face critical challenges that can hinder their performance [142]. The insulating properties of lithium sulfide (Li_2_S) and lithium disulfide (Li_2_S_2_) exacerbate electrochemical polarization and increase impedance, which negatively impacts rate performance. Additionally, the complex conversion process of lithium polysulfides (LiPSs) and the multi-step electron transfer mechanism contribute to sluggish reaction kinetics and reduced cycle life. To enhance the conversion of LiPSs and extend the operational lifespan of Li-S batteries, the development of catalysts with numerous active sites is essential [143]. Nevertheless, the Li/S system encounters several significant limitations that restrict its wider adoption. The insufficient electrochemical performance of sulfur as a cathode material, combined with its insulating characteristics at ambient temperatures, necessitates the addition of substantial conductive additives [144]. Furthermore, lithium polysulfides (Li_2_S_x_, where x = 4, 5, 6, and 8) formed during charging and discharging tend to dissolve in the liquid electrolyte, altering the morphology and chemical composition of the electrodes [143]. The diffusion of active polysulfides introduces notable transport challenges, compromising the cycle performance and Coulombic efficiency of Li/S cells. The formation of insoluble, insulating LixS (where x = 1–2) on the surface can further hinder the accessibility of lithium ions and electrolytes to the sulfur mass at the end of the discharge process. A wealth of research has been dedicated to addressing these challenges.

A study conducted by Shaojun Huang and colleagues focused on developing PPy-coated multi-walled carbon nanotubes (MWCNTs@S-PPy) to enhance the performance of lithium-sulfur batteries [58]. They capitalized on the unique advantages of both MWCNTs and PPy to create composite cathode material. Initially, sulfur was deposited onto the MWCNTs to produce MWCNTs@S, after which PPy was applied to the outer layer, forming MWCNTs@S-PPy. The composite was then vacuum-dried at 155 °C for 12 h. In this innovative configuration, molten sulfur fills the gaps in the MWCNTs, while PPy serves as a conductive matrix. The results from electrochemical impedance spectroscopy (EIS) revealed that MWCNTs@S-PPy exhibited significantly faster electron transfer compared to MWCNTs@S. Furthermore, MWCNTs@S-PPy demonstrated impressive cycling stability and discharge capacity, achieving a remarkable discharge capacity of 1.30 Ah/g at 0.20 C, and maintaining 1.18 Ah/g after 10 cycles. In contrast, the MWCNTs/S cathode displayed subpar electrochemical performance, with an initial discharge capacity of 1.10 Ah/g and only 66% active sulfur utilization, leading to a capacity retention of 55%. The S-PPy-coated MWCNTs exhibited an initial discharge capacity of 1.31 Ah/g with improved active sulfur utilization (75%). After 300 cycles, the reversible capacity reached 0.98 Ah/g, resulting in a capacity retention of 75.6%. These findings strongly indicate that the PPy coating significantly enhances cycle performance and active sulfur utilization compared to the S-coated MWCNTs composite, highlighting its potential as a cathode material for lithium-sulfur batteries.

In another study by Li Fang et al., the authors successfully synthesized a uniform PPy-coated sulfur/graphene aerogel composite using an innovative vapor-phase deposition technique [145]. The PPy layer effectively mitigates polysulfide dissolution through strong chemical interactions, functioning as both a host and adsorbent. Density functional theory simulations further suggested that PPy can trap lithium polysulfides due to its higher bonding energy. This vapor-phase deposition process also enhances the interaction between sulfur and the matrix, promoting high sulfur utilization and favorable rate performance. The resulting PPy@S/GA-VD sample exhibited a discharge capacity of 1.167 Ah/g at 0.20 C and retained a capacity of 0.698 Ah/g after 500 cycles at 0.50 C, demonstrating a minimal degradation rate of just 0.03% per cycle.

Furthermore, in a different study, the PPy-C-coated-sulfur-MWCNT cathode material displayed commendable electrochemical performance at ambient temperature. M. Kazazi et al. explored enhancements in the PPy-C-coated-S-MWCNT composite cathode material through slight modifications to the fabrication process [146]. The electrochemical behavior was assessed at both room and elevated temperatures [146]. In this study, the S/MWCNT composite was synthesized using melt diffusion and liquid-phase infiltration techniques. Initially, raw MWCNTs were heated at 300 °C for one hour to eliminate catalytic impurities and amorphous carbon. The sulfur-coated MWCNT composite was produced by mixing sterilized MWCNTs with sulfur suspension and homogenizing at room temperature, achieving a mass ratio of sulfur to MWCNTs of 70:10, resulting in a powder composite [147]. The PPy-sulfur-coated-MWCNT composite was synthesized via in situ chemical polymerization, coating the surface of the S-coated MWCNT particles. The final PPy/S/MWCNT composite had a weight ratio of 20:70:10. This entire process was conducted in a cold-water bath, and the final product was thoroughly washed and dried. Electrochemical tests on the synthesized (PPy/S/MWCNT) composite cathode material were conducted at varying temperatures (25, 40, and 70 °C) and rates. The findings revealed that temperature exerted a dual influence on the performance of Li-S cells. As the temperature increased, lithium ions moved more efficiently within the cathode structure, enhancing discharge capacity. However, higher temperatures also elevated the dissolution rate of soluble polysulfides into the electrolyte, leading to capacity degradation and shuttle effects. The PPy coating on the S/MWCNT composite effectively retained polysulfides within the cathode structure, minimizing active material loss during cycling, even at elevated temperatures. EIS results demonstrated that the PPy coating reduced the formation of passive layers (Li_2_S and Li_2_S_2_) on the cathode surface, thereby facilitating lithium-ion movement toward active sulfur. As a result, the PPy/S/MWCNT cathode material exhibited enhanced cycle stability and discharge performance.

## 5. Summary and Future Perspectives

To comprehend the PPy-based composites for battery application, this review provides an overall scenario of recent scientific progress on the synthesis of these materials as well as morphological topography. To summarize, there are numerous polymerization methods for the synthesis of PPy-based composites and their significant fabrication conditions and morphological features, which can be beneficial in different types of battery applications. Thus, the design and synthesis of PPy-based composite materials with appropriate polymerization approaches including chemical oxidative, hydrothermal, electrochemical, and epitaxial polymerization, would help to develop numerous composites as cathode materials for high-performance battery applications. This review discussed the recent progress on the synthesis of different PPy-based composites such as PPy/S, PPy/MnOx, MWCNT/PPy, V2)5/PPy, Cl-doped-PPy/rGO, and Fe/α-MnO_2_@PPy composites through different polymerization approach for numerous battery applications. However, significant challenges remain, including low efficiency, short cycle life, and safety concerns, which need to be addressed with more suitable materials. Additionally, issues such as morphological complexity and the development of appropriate cathode materials continue to pose challenges. To address these limitations, future research should focus on the rational design and fabrication of PPy-based composites that combine cost-effectiveness with enhanced functionality. For instance, improving the conductivity and structural stability of PPy through doping or hybridization with synergistic materials could enhance charge transfer kinetics and cycling performance. Addressing morphological complexity, such as volume expansion and structural degradation during repeated cycles, is another critical aspect. This could be achieved by designing advanced architectures like yolk-shell structures, core-shell composites, or hierarchical frameworks that accommodate structural changes without compromising stability. Additionally, developing safer materials through the incorporation of thermally stable components or flame-retardant additives into the PPy matrix can mitigate safety concerns. Enhancing cathode material performance is also essential, which may involve integrating PPy with high-capacity materials like vanadium pentoxide or manganese-based compounds. These approaches collectively aim to overcome existing challenges and pave the way for high-performance, reliable PPy-based batteries.

## Figures and Tables

**Table 1:** Recent reports of the PPy-based nanocomposites synthesized for battery applications.

Nanocomposites	Synthesis Method	Remarks	Applications	Ref.
PPy-sulfur composites	Chemical oxidative	Continuously stirred for 2 h	Lithium-sulfur	[48]
PPy/MnOx nanosheets	Epitaxial polymerization	Reaction took place in liquid phase	Zinc-ion	[49]
Fe/α-MnO_2_@PPy composites	In situ polymerization	Acid-catalyzed polymerization, ultrasonication	Zinc-ion	[30]
MWCNT-PPy nanowire	In situ chemical oxidative	Synthesized at 80 °C for 5 h	Sulfate- and sodium-ion	[50]
Polypyrrole Fe-coated porous Si	Photo-induced polymerization	Synthesized through acid etching from SiAl alloy	Lithium-ion	[51]
β-MnO_2_/PPy composite	Hydrothermal		Zinc-ion	[19]
V_2_O_5_/PPy composite	Hydrothermal	Surface initiated and the reaction mixture was stirred for 24 h	Zinc-ion	[52]
V_2_O_5_-PPy	In situ polymerization	Room temperature synthesis and stirred for 144 h	Zinc-ion	[20]
Oxygen-deficient hydrate vanadium dioxide @ polypyrrole (O_d_−HVO@PPy)	Hydrothermal	One-step process	Zinc-ion	[53]
PPy/MnO_2_/Mn_2_O_3_	In situ polymerization	Ultrasonication and stirring at room temperature	Zinc-ion	[54]
Cl-doped PPy/rGO.	Hydrothermal	Ultrasonic shaking and stirred at 6 h	Sodium-based dual-ion batteries	[31]
LiNi_1/3_Co_1/3_Mn_1/3_O_2_/polypyrrole	Chemical oxidation polymerization	Ultrasound for 20 min	Lithium-ion	[55]
PPy-modified Prussian blue (KHCF@PPy)	In situ polymerization	Magnetic stirring for 4 h	Potassium ion	[56]
MnO/polypyrrole	Hydrothermal	Ultrasonication and stirring for 30 min hours in air	Lithium-sulfur	[57]
MWCNTs@S-PPy	Chemical oxidation polymerization	Sonication at room temperature for 1 h	Lithium-sulfur	[58]
S/PPy	Ball milling process	Stirred for 3 h	Lithium rechargeable	[59]
PPy@S@PPy	Chemical oxidation polymerization	Stirred for 30 min at 0–5 °C	Lithium-sulfur	[60]
V_2_O_5_@PPy	Sol-gel and solvothermal	Vacuum system standing for half an hour at room temperature	Lithium-ion	[61]
PPy-modified GF (graphite felt)	Chemical oxidation polymerization	Room temperature synthesis and reaction time was 24 h	Redox flow	[25]

## Data Availability

Data is contained within the article.

## References

[1] Yuvika S., Varsha S.P., Harish M., Anil K. (2021). A review on synthetic strategies and gas sensing approach for polypyrrole-based hybrid nanocomposites. Polym. Eng. Sci..

[2] Bhadra S., Khastgir D., Singha N.K., Lee J.H. (2009). Progress in preparation, processing and applications of polyaniline. Prog. Polym. Sci..

[3] Chandramika B., Swapan K.D. (2014). Interfacial synthesis of polypyrrole/graphene composites and investigation of their optical, electrical and electrochemical properties. Polym. Int..

[4] Abdirahman Y., Mohammad A.-S., Salah A.-E., Gils A. (2018). Synthesis and Characterization of Conductive Polypyrrole: The Influence of the Oxidants and Monomer on the Electrical, Thermal, and Morphological Properties. Int. J. Polym. Sci..

[5] Ramesan M.T., Santhi V. (2017). In situ synthesis, characterization, conductivity studies of polypyrrole/silver doped zinc oxide nanocomposites and their application for ammonia gas sensing. J. Mater. Sci. Mater. Electron..

[6] Nerkar D.M., Jaware S.E., Padhye G.G. (2016). Fabrication of a novel flexible room temperature hydrogen sulfide (H_2_S) gas sensor based on polypyrrole films. Indian J. Sci. Res..

[7] Yang L., Ying C., Likun Y. (2006). Adjusting the inner structure of polypyrrole nanoparticles through microemulsion polymerization. Mater. Chem. Phys..

[8] Feng Y., Gi X., Fen W. (2002). A flexible giant magnetoresistance sensor prepared completely by electrochemical synthesis. J. Mater. Chem..

[9] Jurewicz K., Delpeux S., Bertagna V., Béguin F., Frackowiak E. (2001). Supercapacitors from nanotubes/polypyrrole composites. Phys. Lett..

[10] Mária O., Miroslava M., Ivan C., Jürgen P., Liane H. (2006). Conductive polypropylene/clay/polypyrrole nanocomposites. Polym. Eng. Sci..

[11] Aditee J., Gangal S.A., Gupta S.K. (2011). Ammonia sensing properties of polypyrrole thin films at room temperature. Sens. Actuators B Chem..

[12] Malook K., Khan M., Ali M. (2019). Polypyrrole-CuO based composites, promotional effects of CuO contents on polypyrrole characteristics. J. Mater. Sci. Mater Electron..

[13] Fatemeh K., Malihe P., Mehdi S.-K., Farhood N. (2017). Morphology control of conducting polypyrrole nanostructures via operational conditions in the emulsion polymerization. J. Appl. Polym. Sci..

[14] Madhurima D., Somenath R. (2021). Polypyrrole and associated hybrid nanocomposites as chemiresistive gas sensors: A comprehensive review. Mater. Sci. Semicond. Process..

[15] Mohd Nurazzi N., Muhammad Harussani M., Siti Zulaikha N.D., Norhana Abdul H., Alinda S., Imran Syakir M., Victor Feizal K., Norli A. (2020). Carbon nanotubes: Functionalisation and their application in chemical sensors. RSC Adv..

[16] Philip G.C., Keith B., Masa I., Zettl A. (2000). Extreme oxygen sensitivity of electronic properties of carbon Nanotubes. Science.

[17] Chowdhury M.S.H., Rahman Khan M.M., Shohag M.R.H., Rahman S., Paul S.K., Rahman M.M., Asiri A.M., Rahman M.M. (2023). Easy synthesis of PPy/TiO_2_/ZnO composites with superior photocatalytic performance, efficient supercapacitors and nitrite sensor. Heliyon.

[18] Chowdhury M.S.H., Rahman Khan M.M., Deb B., Shohag M.R.H., Rahman S., Rahman M.M., Alzahrani K.A., Rahman M.M., Bhuyan M.M., Jeong J.H. (2024). Fabrication of Poly (Ani-co-Py)/NiO Composites with Superb Photocatalytic Performance and Effective p-Nitrophenol Sensor. J. Inorg. Organomet. Polym..

[19] Liao X., Pan C., Pan Y., Yin C. (2021). Synthesis of three-dimensional β-MnO_2_/PPy composite for high-performance cathode in zinc-ion batteries. J. Alloys Compd..

[20] Zhang Y., Huang R., Wang X., Wang Z., Song B., Du Y., Lu Q., Chen X., Sun J. (2022). Facile large-scale preparation of vanadium pentoxide-polypyrrole composite for aqueous zinc-ion batteries. J. Alloys Compd..

[21] Hong X., Liu Y., Li Y., Wang X., Fu J., Wang X. (2020). Application progress of polyaniline, polypyrrole and polythiophene in lithium-sulfur batteries. Polymers.

[22] Hao L., Dong C., Yu D. (2024). Polypyrrole derivatives: Preparation, properties and application. Polymers.

[23] Hao L., Dong C., Zhang L., Zhu K., Yu D. (2022). Polypyrrole nanomaterials: Structure, preparation and application. Polymers.

[24] Chen X., Zhao C., Yang K., Sun S., Bi J., Zhu N., Cai Q., Wang J., Yan W. (2023). Conducting polymers meet lithium–sulfur batteries: Progress, challenges, and perspectives. Energy Environ. Mater..

[25] Ariyamparambil V.J., Kandasubramanian B. (2022). A mini-review on the recent advancement of electrospun MOF-derived nanofibers for energy storage. Chem. Eng. J. Adv..

[26] Suresh Khurd A., Kandasubramanian B. (2022). A systematic review of cellulosic material for green electronics devices. Carbohydr. Polym. Technol. Appl..

[27] Li Q., Dong Q., Zhang T., Xue Z., Li J., Wang Z., Sun H. (2022). Performance of room-temperature activated tubular polypyrrole modified graphite felt composite electrode in vanadium redox flow battery. Electrochim. Acta.

[28] Yadav R., Tirumali M., Wang X., Naebe M., Kandasubramanian B. (2020). Polymer composite for antistatic application in aerospace. Def. Technol..

[29] Duan J., Zou D., Che Z., Weng J., Ji Y., Zhu M., Li A., Zhou P. (2021). A flexible and free-standing Cl−doped PPy/rGO film as cathode material for ultrahigh capacity and long-cycling sodium based dual-ion batteries. Carbon.

[30] Xu J.W., Gao Q.L., Xia Y.M., Lin X.S., Liu W.L., Ren M.M., Kong F.G., Wang S.J., Lin C. (2021). High-performance reversible aqueous zinc-ion battery based on iron-doped alpha-manganese dioxide coated by polypyrrole. J. Colloid Interface Sci..

[31] Duan J., Zou D., Li J., Weng J., Liu Y., Gong S., Li A., Zhou P. (2021). One-dimensional PPy@CNT based on reversible anions doping/dedoping as a novel high-performance cathode for potassium based double ion batteries. Electrochim. Acta.

[32] Zarrabeitia M., Nobili F., Lakuntza O., Carrasco J., Rojo T., Casas-Cabanas M., Muñoz-Márquez M.Á. (2022). Role of the voltage window on the capacity retention of P_2_-Na_2/3_[Fe_1/2_Mn_1/2_]O_2_ cathode material for rechargeable sodium-ion batteries. Commun. Chem..

[33] Xiang H., Deng N., Zhao H., Wang X., Wei L., Wang M., Cheng B., Kang W. (2021). A review on electronically conducting polymers for lithium-sulfur battery and lithium-selenium battery: Progress and prospects. J. Energy Chem..

[34] Kaur G., Kaur A., Kaur H. (2021). Review on nanomaterials/conducting polymer-based nanocomposites for the development of biosen- sors and electrochemical sensors. Polym. Technol. Mater..

[35] Sapana J., Narendra Pal Singh C., Sampath C., Radhamanohar A., Manda S., Narendra Singh C., Abbas R. (2023). A short review on conducting polymer nanocomposites. Biomed. Mater. Devices.

[36] Rahman Khan M.M., Wee Y.K., Mahmood W.A.K. (2012). Effects of CuO on the morphology and conducting properties of PANI nanofibers. Synth. Met..

[37] Rahman Khan M.M., Wee Y.K., Mahmood W.A.K. (2015). Synthesis of PANI-CaO composite nanofibers with controllable diameter and electrical conductivity. Polym. Compos..

[38] Rahman Khan M.M., Pal S., Hoque M.M., Alam M.R., Younus M., Kobayashi H. (2019). Simple fabrication of PVA–ZnS composite films with superior photocatalytic performance: Enhanced luminescence property, morphology, and thermal stability. ACS Omega.

[39] An K.H., Jeong S.Y., Hwang H.R., Lee Y.H. (2004). Enhanced sensitivity of a gas sensor incorporating single-walled carbon nanotube–polypyrrole nanocomposites. Advanc. Mater..

[40] Sharma R.K., Rastogi A.C., Desu S.B. (2008). Manganese oxide embedded polypyrrole nanocomposites for electrochemical superca-pacitor. Electrochim. Acta.

[41] Rahman Khan M.M., Islam M., Amin M.K., Paul S.K., Rahman S., Talukder M.M., Rahman M.M. (2022). Simplistic fabrication of aniline and pyrrole-based poly (Ani-co-Py) for efficient photocatalytic performance and supercapacitors. Int. J. Hydrogen Energy.

[42] Kaplin D.A., Qutubuddin S. (1995). Electrochemically synthesized polypyrrole films: Effects of polymerization potential and electrolyte type. Polymer.

[43] Kumar D., Sharma R.C. (1998). Advances in conductive polymers. Eur. Polym. J..

[44] Zhou X.J., Harmer A.J., Heinig N.F., Leung K.T. (2004). Parametric study on electrochemical deposition of copper nanoparticles on an ultrathin polypyrrole film deposited on a gold film electrode. Langmuir.

[45] Xuan J., Min H., Lu C., Zhang M., Li R., Gao L., Fu F., Liang Z. (2019). Three-dimensional carambola-like MXene/polypyrrole composite produced by one-step co-electrodeposition method for electrochemical energy storage. Electrochim. Acta.

[46] Zhu M., Huang Y., Deng Q., Zhou J., Pei Z., Xue Q., Huang Y., Wang Z., Li H., Huang Q. (2016). Highly flexible, freestanding supercapacitor electrode with enhanced performance obtained by hybridizing polypyrrole chains with mxene. Adv. Energy Mater..

[47] Hassan M., Rawat R., Lee P., Hassan S., Qayyum A., Ahmad R., Murtaza G., Zakaullah M. (2008). Synthesis of nanocrystalline multiphase titanium oxycarbide (TiC_x_O_y_) thin films by UNU/ICTP and NX2 plasma focus devices. Appl. Phys. A.

[48] Veronika N., Alexandra G., Ondrej P., Haojie F., Miroslav A., Andrea S. (2024). Investigation of polypyrrole based composite material for lithium sulfur batteries. Sci. Rep..

[49] Zang X., Wang X., Liu H., Ma X., Wang W., Ji J., Chen J., Li R., Xue M. (2020). Enhanced ion conduction via epitaxially polymerized two-dimensional conducting polymer for high-performance cathode in zinc-ion batteries. ACS Appl. Mater. Interfaces.

[50] Lim H., Jung J.H., Park Y.M., Lee H.-N., Kim H.-J. (2018). High-performance aqueous rechargeable sulfate- and sodium-ion battery based on polypyrrole-MWCNT core-shell nanowires and Na_0.44_MnO_2_ nanorods. Appl. Surf. Sci..

[51] Xu Z., Zheng E., Xiao Z., Shao H., Liu Y., Wang J. (2023). Photo-Initiated in situ synthesis of polypyrrole Fe-Coated porous silicon microspheres for High-performance Lithium-ion battery anodes. Chem. Eng. J..

[52] Qin X., Wang X., Sun J., Lu Q., Omar A., Mikhailova D. (2020). Polypyrrole wrapped V_2_O_5_ nanowires composite for advanced aqueous zinc-ion batteries. Front. Energy Res..

[53] Zhang Z., Xi B., Wang X., Ma X., Chen W., Feng J., Xiong S. (2021). Oxygen defects engineering of VO_2_·*X*H_2_O nanosheets via in situ polypyrrole polymerization for efficient aqueous zinc ion storage. Adv. Funct. Mater..

[54] Huang A., Zhou W., Wang A., Chen M., Chen J., Tian Q., Xu J. (2021). Self-initiated coating of polypyrrole on MnO_2_/Mn_2_O_3_ nanocomposite for high-performance aqueous zinc-ion batteries. Appl. Surf. Sci..

[55] Zhu L., Xie L., Bao C., Yan X., Cao X. (2019). LiNi_1/3_Co_1/3_Mn_1/3_O_2_/polypyrrole composites as cathode materials for high-performance lithium-ion batteries. Int. J. Energy Res..

[56] Xue Q., Li L., Huang Y., Huang R., Wu F., Chen R. (2019). Polypyrrole-modified prussian blue cathode material for potassium ion batteries via in situ polymerization coating. ACS Appl. Mater. Interfaces.

[57] Feng Y., Liu H., Lu Q., Liu Y., Li J., He X., Liu X., Mikhailova D. (2022). Designing hierarchical MnO/polypyrrole heterostructures to couple polysulfides adsorption and electrocatalysis in lithium-sulfur batteries. J. Power Sources.

[58] Huang S., Wang X., Hu R., Wang X., Yang X., Zhao N., Lei W., Zhu L., Peng J. (2020). Polypyrrole-S-coated MWCNT composites as cathode materials for lithium-sulfur batteries. Ionics.

[59] Zhang Y., Bakenov Z., Zhao Y., Konarov A., Doan T.N.L., Malik M., Paron T., Chen P. (2012). One-step synthesis of branched sulfur/polypyrrole nanocomposite cathode for lithium rechargeable batteries. J. Power Sources.

[60] Liang X., Zhang M., Kaiser M.R., Gao X., Konstantinov K., Tandiono R., Wang Z., Liu H.-K., Dou S.-X., Wang J. (2015). Split-half-tubular polypyrrole@sulfur@polypyrrole composite with a novel three-layer-3D structure as cathode for lithium/sulfur batteries. Nano Energy.

[61] Liang X., Gao G., Jiang X., Zhang W., Bi W., Wang J., Du Y., Wu G. (2020). Preparation of hydrophobic ppy coated v_2_o_5_ yolk–shell nanospheres-based cathode materials with excellent cycling performance. ACS Appl. Energy Mater..

[62] Sood Y., Singha K., Mudila H., Lokhande P.E., Singh L., Kumar D., Kumar A., Mubarak N.M., Hadi M. (2024). DehghaniInsights into properties, synthesis and emerging applications of polypyrrole-based composites, and future prospective: A review. Heliyon.

[63] Pang A.L., Arsad A., Ahmadipour M. (2021). Synthesis and factor affecting on the conductivity of polypyrrole: A short review. Polym. Adv. Technol..

[64] Reese C.J., Boyes S.G. (2021). Progress in Polymer Science New methods in polymer brush synthesis: Non-vinyl-based semiflexible and rigid-rod polymer brushes. Prog. Polym. Sci..

[65] Song H., Li T., Han Y., Wang Y., Zhang C., Wang Q. (2016). Optimizing the polymerization conditions of conductive polypyrrole. J. Photopolym. Sci. Technol..

[66] Saad A., Cabet E., Lilienbaum A., Hamadi S., Abderrabba M., Chehimi M.M. (2017). Polypyrrole/Ag/mesoporous silica nanocomposite particles: Design by photopolymerization in aqueous medium and antibacterial activity. J. Taiwan Inst. Chem. Eng..

[67] Jlassi K., Sliem M.H., Benslimane F.M., Eltai N.O., Abdullah A.M. (2020). Design of hybrid clay/polypyrrole decorated with silver and zinc oxide nanoparticles for anticorrosive and antibacterial applications. Prog. Org. Coat..

[68] Heydarnezhad H.R., Pourabbas B., Tayefi M. (2018). Conducting electroactive polymers via photopolymerization: A review on synthesis and applications. Polym. Plast. Technol. Eng..

[69] Chavan U.D., Prajith P., Kandasubramanian B. (2022). Polypyrrole based cathode material for battery application. Chem. Eng. J. Adv..

[70] Lawal A.T., Wallace G.G. (2014). Vapour phase polymerisation of conducting and non-conducting polymers: A review. Talanta.

[71] Tung T.T., Castro M., Feller J.-F., Kim T.Y., Suh K.S. (2013). Hybrid film of chemically modified graphene and vapor-phase-polymerized PEDOT for electronic nose applications. Org. Electron..

[72] Mohammadi A., Lundström I., Salaneck W.R., Inganäs O. (1986). Polypyrrole pre-pared by chemicalvapour deposition using hydrogen peroxide and hydrochloric acid. Synth. Met..

[73] Savage N.O. (2009). Gas sensing composites of metal oxides with vapor-deposited polypyrrole. Sens. Actuator B-Chem..

[74] Winther-Jensen B., Krebs F.C. (2006). High-conductivity large-area semi-transparent electrodes for polymer photovoltaics by silk screen printing and vapour-phase deposition. Sol. Energy Mater. Sol. Cells.

[75] Cho J., Shin K.-H., Jang J. (2010). Micropatterning of conducting polymer tracks on plasma treated exible substrate using vapor-phase polymerization mediated inkjet printing. Synth. Met..

[76] Cho M.S., Kim S.Y., Nam J.D., Lee Y. (2008). Preparation of PEDOT/Cu composite by in situ redox reaction between EDOT and copper(II) chloride. Synth. Met..

[77] Cao J., Chen C., Zhao Q., Zhang N., Lu Q., Wang X., Niu Z., Chen J. (2016). A flexible nanostructured paper of a reduced graphene oxide-sulfur composite for high-performance lithium-sulfur batteries with unconventional configurations. Adv. Mater..

[78] Zhang D., Cai R., Zhou Y., Shao Z., Liao X.-Z., Ma Z.-F. (2010). Effect of milling method and time on the properties and electrochemical performance of LiFePO_4_/C composites prepared by ball milling and thermal treatment. Electrochim. Acta.

[79] Adekoya G.J., Adekoya O.C., Sadiku R.E., Hamam Y., Ray S.S. (2022). Applications of MXene-Containing Polypyrrole Nanocomposites in Electrochemical Energy Storage and Conversion. ACS Omega.

[80] Li C., Zhang K., Cheng X., Li J., Jiang Y., Li P., Wang B., Peng H. (2023). Polymers for flexible energy storage devices. Prog. Polym. Sci..

[81] Wang T., Chen S., Chen K.-J. (2023). Metal-Organic Framework Composites and Their Derivatives as Efficient Electrodes for Energy Storage Applications: Recent Progress and Future Perspectives. Chem. Rec..

[82] Choudhary R.B., Ansari S., Purty B. (2020). Robust electrochemical performance of polypyrrole (PPy) and polyindole (PIn) based hybrid electrode materials for supercapacitor application: A review. J. Energy Storage.

[83] Zhao F., Shi Y., Pan L., Yu G. (2017). Multifunctional Nanostructured Conductive Polymer Gels: Synthesis, Properties, and Applications. Acc. Chem. Res..

[84] Hao X., Sun H., Ren Z., Huang Z., Xu Y., Li J. (2024). Recent advances in zinc sulfide-based anode regulation strategy for Na-ion batteries. J. Energy Storage.

[85] Ruan Y., Chen L., Cui L., An Q. (2022). PPy-Modified Prussian Blue Cathode Materials for Low-Cost and Cycling-Stable Aqueous Zinc-Based Hybrid Battery. Coatings.

[86] Puthirath A.B., Baburaj A., Kato K., Salpekar D., Chakingal N., Cao Y., Babu G., Ajayan P.M. (2019). High sulfur content multifunctional conducting polymer composite electrodes for stable Li-S battery. Electrochim. Acta.

[87] Zeng Z., Liu X. (2018). Sulfur Immobilization by “Chemical Anchor” to Suppress the Diffusion of Polysulfides in Lithium–Sulfur Batteries. Adv. Mater. Interfaces.

[88] Zhou G., Xu L., Hu G., Mai L., Cui Y. (2019). Nanowires for Electrochemical Energy Storage. Chem. Rev..

[89] Li M., Hicks R.P., Chen Z., Luo C., Guo J., Wang C., Xu Y. (2023). Electrolytes in Organic Batteries. Chem. Rev..

[90] Li L., Duan Y. (2023). Engineering Polymer-Based Porous Membrane for Sustainable Lithium-Ion Battery Separators. Polymers.

[91] Long L., Wang S., Xiao M., Meng Y. (2016). Polymer electrolytes for lithium polymer batteries. J. Mater. Chem. A.

[92] Bubulinca C., Kazantseva N.E., Pechancova V., Joseph N., Fei H., Venher M., Ivanichenko A., Saha P. (2023). Development of All-Solid-State Li-Ion Batteries: From Key Technical Areas to Commercial Use. Batteries.

[93] Hu A., Zhou M., Lei T., Hu Y., Du X., Gong C., Shu C., Long J., Zhu J., Chen W. (2020). Optimizing Redox Reactions in Aprotic Lithium–Sulfur Batteries. Adv. Energy Mater..

[94] Alam S., Jadoon S., Iqbal M.Z., Hegazy H.H., Ahmad Z., Yahia I.S. (2024). Recent progress in polypyrrole and its composites with carbon, metal oxides, sulfides and other conducting polymers as an emerging electrode material for asymmetric supercapacitors. J. Energy Storage.

[95] Rumon M.M.H., Sarkar S.D., Alam M.M., Roy C.K. (2023). Nanomaterials for Self-Healing Hydrogels. Emerg. Appl. Nanomater..

[96] Deng J., Luo W.-B., Chou S.-L., Liu H.-K., Dou S.-X. (2018). Sodium-Ion Batteries: From Academic Research to Practical Commercialization. Adv. Energy Mater..

[97] Zhao L., Zhang T., Li W., Li T., Zhang L., Zhang X., Wang Z. (2023). Engineering of Sodium-Ion Batteries: Opportunities and Challenges. Engineering.

[98] Chayambuka K., Mulder G., Danilov D.L., Notten P.H.L. (2018). Sodium-Ion Battery Materials and Electrochemical Properties Reviewed. Adv. Energy Mater..

[99] Chen Z., Wu X., Sun Z., Pan J., Han J., Wang Y., Liu H., Shen Y., Li J., Peng D.-L. (2024). Enhanced Fast-Charging and Longevity in Sodium-Ion Batteries through Nitrogen-Doped Carbon Frameworks Encasing Flower-Like Bismuth Microspheres. Adv. Energy Mater..

[100] Gong D., Wei C., Liang Z., Tang Y. (2021). Recent Advances on Sodium-Ion Batteries and Sodium Dual-Ion Batteries: State-of-the-Art Na+ Host Anode Materials. Small Sci..

[101] Liu Y., Qiu M., Hu X., Yuan J., Liao W., Sheng L., Chen Y., Wu Y., Zhan H., Wen Z. (2023). Anion Defects Engineering of Ternary Nb-Based Chalcogenide Anodes Toward High-Performance Sodium-Based Dual-Ion Batteries. Nano-Micro Lett..

[102] Qiao X., Chen T., He F., Li H., Zeng Y., Wang R., Yang H., Yang Q., Wu Z., Guo X. (2024). Solvation Effect: The Cornerstone of High-Performance Battery Design for Commercialization-Driven Sodium Batteries. Small.

[103] Guo Z., Xu Z., Xie F., Feng J., Titirici M. (2021). Strategies for High Energy Density Dual-Ion Batteries Using Carbon-Based Cathodes. Adv. Energy Sustain. Res..

[104] Zhao Z., Alshareef H.N. (2024). Sustainable Dual-Ion Batteries beyond Li. Adv. Mater..

[105] Rumon M.M.H., Sarkar S.D., Uddin M.M., Alam M.M., Karobi S.N., Ayfar A., Azam M.S., Roy C.K. (2022). Graphene oxide based crosslinker for simultaneous enhancement of mechanical toughness and self-healing capability of conventional hydrogels. RSC Adv..

[106] Thakur K., Kandasubramanian B. (2019). Graphene and Graphene Oxide-Based Composites for Removal of Organic Pollutants: A Review. J. Chem. Eng. Data.

[107] Chen X., Liu L., Yan Z., Huang Z., Zhou Q., Guo G., Wang X. (2016). The excellent cycling stability and superior rate capability of polypyrrole as the anode material for rechargeable sodium ion batteries. RSC Adv..

[108] Jiang Y., Lao J., Dai G., Ye Z. (2024). Advanced Insights on MXenes: Categories, Properties, Synthesis, and Applications in Alkali Metal Ion Batteries. ACS Nano.

[109] Weng J., Duan J., Sun C., Liu P., Li A., Zhou P., Zhou J. (2020). Construction of hierarchical K_0.7_Mn_0.7_Mg_0.3_O_2_ microparticles as high capacity & long cycle life cathode materials for low-cost potassium-ion batteries. Chem. Eng. J..

[110] Jain V., Kandasubramanian B. (2020). Functionalized graphene materials for hydrogen storage. J. Mater. Sci..

[111] Purabgola A., Mayilswamy N., Kandasubramanian B. (2022). Graphene-based TiO_2_ composites for photocatalysis & environmental remediation: Synthesis and progress. Environ. Sci. Pollut. Res..

[112] Li T., Zhang Q. (2018). Advanced metal sulfide anode for potassium ion batteries. J. Energy Chem..

[113] Huang Y., Xie M., Zhang J., Wang Z., Jiang Y., Xiao G., Li S., Li L., Wu F., Chen R. (2017). A novel border-rich Prussian blue synthetized by inhibitor control as cathode for sodium ion batteries. Nano Energy.

[114] Jiang Y., Yu S., Wang B., Li Y., Sun W., Lu Y., Yan M., Song B., Dou S. (2016). Prussian Blue@C Composite as an Ultrahigh-Rate and Long-Life Sodium-Ion Battery Cathode. Adv. Funct. Mater..

[115] Li G., Sun L., Zhang S., Zhang C., Jin H., Davey K., Liang G., Liu S., Mao J., Guo Z. (2024). Developing Cathode Materials for Aqueous Zinc Ion Batteries: Challenges and Practical Prospects. Adv. Funct. Mater..

[116] Chen M., Liu Q., Wang S.-W., Wang E., Guo X., Chou S.-L. (2019). High-Abundance and Low-Cost Metal-Based Cathode Materials for Sodium-Ion Batteries: Problems, Progress, and Key Technologies. Adv. Energy Mater..

[117] Kamenskii M.A., Volkov F.S., Eliseeva S.N., Tolstopyatova E.G., Kondratiev V.V. (2023). Enhancement of Electrochemical Performance of Aqueous Zinc Ion Batteries by Structural and Interfacial Design of MnO_2_ Cathodes: The Metal Ion Doping and Introduction of Conducting Polymers. Energies.

[118] Alfaruqi M.H., Islam S., Mathew V., Song J., Kim S., Tung D.P., Jo J., Kim S., Baboo J.P., Xiu Z. (2017). Ambient redox synthesis of vanadium-doped manganese dioxide nanoparticles and their enhanced zinc storage properties. Appl. Surf. Sci..

[119] Liu Y., Chi X., Han Q., Du Y., Huang J., Liu Y., Yang J. (2019). α-MnO_2_ nanofibers/carbon nanotubes hierarchically assembled microspheres: Approaching practical applications of high-performance aqueous Zn-ion batteries. J. Power Sources.

[120] Dong R., Zhang T., Liu J., Li H., Hu D., Liu X., Xu Q. (2022). Mechanistic Insight into Polypyrrole Coating on V_2_O_5_ Cathode for Aqueous Zinc-Ion Battery. ChemElectroChem.

[121] Barbosa J.C., Gonçalves R., Costa C.M., Lanceros-Mendez S. (2021). Recent Advances on Materials for Lithium-Ion Batteries. Energies.

[122] Nzereogu P.U., Omah A.D., Ezema F.I., Iwuoha E.I., Nwanya A.C. (2022). Anode materials for lithium-ion batteries: A review. Appl. Surf. Sci. Adv..

[123] Landi B.J., Ganter M.J., Cress C.D., DiLeo R.A., Raffaelle R.P. (2009). Carbon nanotubes for lithium ion batteries. Energy Environ. Sci..

[124] Zhu S.M., Zhou H.S., Hibino M., Honma I., Ichihara M. (2005). Synthesis of MnO_2_ Nanoparticles Confined in Ordered Mesoporous Carbon Using a Sonochemical Method. Adv. Funct. Mater..

[125] Lepage D., Michot C., Liang G., Gauthier M., Schougaard S.B. (2011). A soft chemistry approach to coating of LiFePO_4_ with a conducting polymer. Angew. Chem. Int. Ed..

[126] Hammond G.P., Hazeldine T. (2015). Indicative energy technology assessment of advanced rechargeable batteries. Appl. Energy.

[127] Cui L., Shen J., Cheng F., Tao Z., Chen J. (2011). SnO_2_ nanoparticles@polypyrrole nanowires composite as anode materials for rechargeable lithium-ion batteries. J. Power Sources.

[128] Liu R., Li D., Wang C., Li N., Li Q., Lü X., Spendelow J.S., Wu G. (2014). Core–shell structured hollow SnO_2_–polypyrrole nanocomposite anodes with enhanced cyclic performance for lithium-ion batteries. Nano Energy.

[129] Castro Neto A.H., Guinea F., Peres N.M.R., Novoselov K.S., Geim A.K. (2009). The electronic properties of graphene. Rev. Mod. Phys..

[130] Zhao Y., Li J., Wang N., Wu C., Dong G., Guan L. (2012). Fully Reversible Conversion between SnO_2_ and Sn in SWNTs@SnO_2_@PPy Coaxial Nanocable As High Performance Anode Material for Lithium Ion Batteries. J. Phys. Chem. C.

[131] Wang J.-G., Sun H., Liu H., Jin D., Liu X., Li X., Kang F. (2018). Triaxial Nanocables of Conducting Polypyrrole@SnS2@Carbon Nanofiber Enabling Significantly Enhanced Li-Ion Storage. ACS Appl. Mater. Interfaces.

[132] He Q., Rui K., Chen C., Yang J., Wen Z. (2017). Interconnected CoFe_2_O_4_–Polypyrrole Nanotubes as Anode Materials for High Performance Sodium Ion Batteries. ACS Appl. Mater. Interfaces.

[133] Thakkar P., Khatri S., Dobariya D., Patel D., Dey B., Singh A.K. (2024). Advances in materials and machine learning techniques for energy storage devices: A comprehensive review. J. Energy Storage.

[134] Gilbert M., Kandasubramanian B. (2003). Nickel coated mica for conductive compounds. Macromol. Symp..

[135] Shin M.-R., Son J.-T. (2017). Optimization of PPy@LiNi_0.6_Co_0.2_Mn_0.2_O_2_ composite to achieve high electrochemical performance. J. Nanosci. Nanotechnol..

[136] Xu L., Guo P., He H., Zhou N., Ma J., Wang G., Zhang C., Su C. (2017). Preparation of TEMPO-contained pyrrole copolymer by in situ electrochemical polymerization and its electrochemical performances as cathode of lithium ion batteries. Ionics.

[137] Sun X., Zhang H., Zhou L., Huang X., Yu C. (2016). Polypyrrole-Coated Zinc Ferrite Hollow Spheres with Improved Cycling Stability for Lithium-Ion Batteries. Small.

[138] Sun L., Huang X., Li Y., Deng L., Mi H., Ren X., Zhang P. (2021). Controlled synthesis and lithium storage performance of NiCo_2_O_4_/PPy composite materials. J. Phys. Chem. Solids.

[139] Jan W., Khan A.D., Iftikhar F.J., Ali G. (2023). Recent advancements and challenges in deploying lithium sulfur batteries as economical energy storage devices. J. Energy Storage.

[140] Yao W., Liao K., Lai T., Sul H., Manthiram A. (2024). Rechargeable Metal-Sulfur Batteries: Key Materials to Mechanisms. Chem. Rev..

[141] Lee B.-J., Kang T.-H., Lee H.-Y., Samdani J.S., Jung Y., Zhang C., Yu Z., Xu G.-L., Cheng L., Byun S. (2020). Revisiting the Role of Conductivity and Polarity of Host Materials for Long-Life Lithium–Sulfur Battery. Adv. Energy Mater..

[142] Su D., Zhou D., Wang C., Wang G. (2018). Toward High Performance Lithium–Sulfur Batteries Based on Li2S Cathodes and Beyond: Status, Challenges, and Perspectives. Adv. Funct. Mater..

[143] Wu F., Chen J., Li L., Zhao T., Liu Z., Chen R. (2013). Polyethylene-Glycol-Doped Polypyrrole Increases the Rate Performance of the Cathode in Lithium–Sulfur Batteries. ChemSusChem.

[144] Rehman S., Khan K., Zhao Y., Hou Y. (2017). Nanostructured cathode materials for lithium–sulfur batteries: Progress, challenges and perspectives. J. Mater. Chem. A.

[145] Li F., Kaiser M.R., Ma J., Hou Y., Zhou T., Han Z., Lai W., Chen J., Guo Z., Liu H. (2020). Uniform Polypyrrole Layer-Coated Sulfur/Graphene Aerogel via the Vapor-Phase Deposition Technique as the Cathode Material for Li–S Batteries. ACS Appl. Mater. Interfaces.

[146] Kazazi M., Vaezi M.R., Kazemzadeh A. (2014). Enhanced rate performance of polypyrrole-coated sulfur/MWCNT cathode material: A kinetic study by electrochemical impedance spectroscopy. Ionics.

[147] Li X., Cao Y., Qi W., Saraf L.V., Xiao J., Nie Z., Mietek J., Zhang J.-G., Schwenzer B., Liu J. (2011). Optimization of mesoporous carbon structures for lithium–sulfur battery applications. J. Mater. Chem..

